# Reductions in endometriosis-associated pain among women treated with elagolix are consistent across a range of baseline characteristics reflective of real-world patients

**DOI:** 10.1186/s12905-021-01385-3

**Published:** 2021-06-16

**Authors:** Mauricio S. Abrao, Eric Surrey, Keith Gordon, Michael C. Snabes, Hui Wang, Horia Ijacu, Hugh S. Taylor

**Affiliations:** 1 Endometriosis Section, Gynecologic Division, Hospital das Clinicas HCFMUSP, Faculdade de Medicina, Universidade de Sao Paulo, Rua Sao Sebastiao 550, São Paulo, SP 04708-000 Brazil; 2 Gynecologic Division, Hospital BP-A Beneficencia Portuguesa de Sao Paulo, São Paulo, SP Brazil; 3 Colorado Center for Reproductive Medicine, Lone Tree, CO USA; 4 Departments of Clinical Development, Medical Affairs, and Statistics, AbbVie Inc., North Chicago, IL USA; 5 Department of Obstetrics, Gynecology and Reproductive Sciences, Yale School of Medicine, New Haven, CT USA

**Keywords:** Dysmenorrhea, Dyspareunia, Endometriosis, Gonadotropin-releasing hormone, Health-related quality of life

## Abstract

**Background:**

Elagolix is an oral, gonadotropin-releasing hormone (GnRH) receptor antagonist, that significantly reduces dysmenorrhea and non-menstrual pelvic pain (NMPP) in women with moderate to severe endometriosis-associated pain.

**Methods:**

Data were pooled from two 6-month, placebo-controlled, phase 3 studies (Elaris Endometriosis [EM]-I and II) in which 2 doses of elagolix were evaluated (150 mg once daily and 200 mg twice daily). Pooled data from > 1600 women, aged 18–49, were used to evaluate the efficacy of elagolix and health-related quality of life (HRQoL) in prespecified subgroups of women with various baseline characteristics.

**Results:**

Of the 1686 women treated, 1285 (76.2%) completed the studies. The percentages of women with clinically meaningful reductions in dysmenorrhea and NMPP were generally consistent by subgroup. Significant treatment by subgroup interaction was demonstrated for dysmenorrhea response in baseline analgesic use (*p* < 0.01) and previous history of pregnancy (*p* < 0.05) subgroups, and for NMPP response in the baseline NMPP score (*p* < 0.05) and history of pregnancy (*p* < 0.05) subgroups. Patient-reported reduction in pain at month 3 was significant across all subgroups taking elagolix 200 mg BID, and significant across most subgroups with elagolix 150 mg QD. Women across subgroups experienced improvement within each domain of the Endometriosis Health Profile-30 (EHP-30), although significant treatment by subgroup interactions were observed in several categories.

**Conclusions:**

Elagolix was effective in reducing dysmenorrhea and NMPP, and improving HRQoL, compared with placebo across numerous subgroups of women with various baseline characteristics, covering a broad segment of the endometriosis disease and patient types.

*Clinical trial registration*: ClinicalTrials.gov: https://www.clinicaltrials.gov/ct2/show/NCT01620528; https://www.clinicaltrials.gov/ct2/show/NCT01931670.

**Supplementary Information:**

The online version contains supplementary material available at 10.1186/s12905-021-01385-3.

## Introduction

Endometriosis is a chronic, estrogen-dependent, inflammatory condition that is a frequently encountered gynecologic disease, with a 6–10% incidence rate in women of reproductive age [1, 2]. The condition is characterized by the implantation of endometrial-like tissue outside the uterus with development, progression/regression, and active remodeling resulting in several different types of lesions [3]. While the pathophysiology of endometriosis is not yet fully understood, accumulating evidence suggests endometriosis may be driven by alterations to the peritoneal microenvironment caused by immune cells, adhesion molecules, extracellular matrix metalloproteinases, and pro-inflammatory cytokines, which creates conditions for ectopic endometrial cells to differentiate, adhere, survive, and proliferate [4–6]. Many factors are associated with risk of endometriosis, including age, body mass index (BMI), race, ethnicity, history of pregnancy, oral contraceptive use, and parity [7–14].

Although endometriosis can be asymptomatic, women often experience a range of symptoms with dysmenorrhea, non-menstrual pelvic pain (NMPP), and dyspareunia being more common; other symptoms include constipation, dyschezia, dysuria, and infertility [1]. For many women, the burden of symptoms associated with endometriosis also reduces quality of life and profoundly affects psychological well-being, resulting in depression and anxiety [15–17].

Medical therapy may reduce the systemic effects of endometriosis [18]. Current treatment options include non-steroidal anti-inflammatory drugs (NSAIDs), progestin-containing contraceptives, and injectable depot formulations of gonadotropin-releasing hormone (GnRH) agonists. Although GnRH agonists are effective, initial hormonal flare effects may make symptoms worse [19], and side effects, including progressive bone loss and severe vasomotor symptoms due to the reduction of estrogen levels to post-menopausal levels, are experienced with long-term use [2, 20, 21].

Elagolix, an oral GnRH antagonist, approved in the United States, Canada, and Israel for the treatment of endometriosis-associated pain, provides an alternative rapid, reversible, and dose-dependent treatment for patients. Two phase 3, double-blind, randomized, placebo-controlled multicenter studies, Elaris Endometriosis I (EM-I) and Elaris Endometriosis II (EM-II), were conducted in women aged 18–49 years in North America and globally who were surgically diagnosed as having endometriosis with moderate or severe endometriosis-associated pain. Results from these studies demonstrate a statistically significant increase in the proportion of responders (controlled for rescue analgesic use) with a clinically meaningful reduction (determined by receiver operating characteristics analysis) of dysmenorrhea and NMPP at month 3 based on daily scores recorded in an electronic diary in women treated with elagolix 150 mg once a day (QD) or 200 mg twice a day (BID), or placebo [22].

Medical therapy of endometriosis-associated pain is often the first-line treatment [23]. When developing a medical treatment plan for endometriosis, multiple factors are taken into consideration, such as the severity of disease and patient symptoms [23]. However, there is a dearth of research on factors associated with response to medical therapy.

Given the many factors that play a role in the risk of endometriosis and treatment response, data were pooled from EM-I and EM-II and prespecified subgroups were analyzed to determine the efficacy of elagolix in treating endometriosis-associated pain in women with various baseline characteristics, including, among others, age, BMI, time since diagnosis, and measures of baseline disease severity. Further, measures of health-related quality of life (HRQoL) were analyzed by subgroup to evaluate the effects of elagolix on the HRQoL of women with endometriosis who have various baseline demographic and clinical characteristics.

## Methods

### Study design

Details of the overall study designs were published previously by Taylor et al. [22]. Briefly, premenopausal women (aged 18–49 years) who were surgically diagnosed with endometriosis within the past 10 years were enrolled in the study. Women were excluded if they had a bone mineral density (BMD) *z* score of the lumbar spine, femoral neck, or total hip less than − 1.5 at screening, or had clinically significant gynecologic conditions other than endometriosis (including adenomyosis). Each study consisted of a washout period of hormone therapies (if applicable), a screening period (up to 75 days including 2 menstrual cycles and during which dysmenorrhea and NMPP scores were assessed), and a 6-month treatment period [22]. Women then entered a post-treatment follow-up period of up to 12 months or a 6-month extension study (EM-III and EM-IV) [24]. Eligible women who completed the washout and screening periods were randomized in a ratio of 3:2:2 to receive placebo, elagolix 150 mg QD, or elagolix 200 mg BID over the 6-month treatment period. Only results from the pooled EM-I and EM-II initial 6-month treatment period will be reported here.

These studies were conducted in accord with the International Council for Harmonisation, Good Clinical Practice guidelines, the ethical concepts of the Declaration of Helsinki, and/or all applicable federal and local regulations and oversight of an institutional review board.

### Efficacy endpoints

Co-primary efficacy endpoints for EM-I and EM-II were the proportion of women at month 3 with both stable or decreased rescue analgesic use and a clinically meaningful reduction of dysmenorrhea and NMPP. As previously described, clinically meaningful reductions were determined using receiver operating characteristics analysis to determine a threshold in pain reduction for each type of pain as measured by the daily assessment of endometriosis pain (using an electronic 4-point pain impact scale completed daily by the participants). Further details describing the determination of clinically meaningful reductions in dysmenorrhea (≥ 0.81 in EM-I and ≥ 0.85 in EM-II from baseline) and NMPP (≥ 0.36 in EM-I and ≥ 0.43 in EM-II from baseline) have been published previously [22]. Responder rates for dysmenorrhea and NMPP were also assessed at month 6 to demonstrate efficacy persistence during the treatment period.

Prespecified subgroup analyses for the co-primary endpoints were performed for the following demographic and disease severity subgroups: (1) age (years: < 25, 25 to 35, > 35), BMI (kg/m^2^: < 25, 25 to ≤ 29.9, ≥ 30), race (Black, White, Other), ethnicity (Hispanic/Latino, Other), and (2) the following disease severity subgroups: baseline dysmenorrhea score (< 2.17 [baseline median], ≥ 2.17), baseline NMPP score (< 1.54 [baseline median], ≥ 1.54), baseline dyspareunia score (< 1.40 [baseline median], ≥ 1.40), time since diagnosis (years: < 2, 2 to < 5, ≥ 5), analgesic use at baseline (none, opioid, NSAID, or both), previous use of GnRH analog therapy (includes the use of both agonists and antagonists [“yes” or “no”]), previous use of hormonal treatment before entering a washout period (required for those women using hormonal therapy [“yes” or “no”]), and history of pregnancy (“yes” or “no”).

### Measures of health-related quality of life

Each participant received an electronic patient-recorded outcome device (e-Diary [e-PRO, Medable, Inc, Palo Alto, CA]). Throughout the screening and treatment periods, women used the e-Diary to report assessments of dysmenorrhea, NMPP, dyspareunia, uterine bleeding, and the use of allowed analgesic medications for endometriosis-associated pain; the e-Diary was also used to record numeric rating scale (NRS) scores. The NRS was used to measure endometriosis-related pain with and without menstruation. Women were instructed to choose the number (0 [no pain] to 10 [worst possible]) that best described their endometriosis pain over the last 24 h at its worst.

The Patient Global Impression of Change (PGIC) scale [25] was used monthly to evaluate the change in endometriosis-related pain from initiation of the treatment period; this measure asks women to choose 1 of 7 responses (1 = very much improved, 7 = very much worse). Women also provided responses to the Endometriosis Health Profile-30 (EHP-30) [26] self-administered questionnaire. The studies used both the core EHP-30 items (pain, control and powerlessness, emotional well-being, social support, and self-image), and modular section C (sexual relationship domain) [27].

### Safety evaluations

Safety evaluations were previously described for EM-I and EM-II and the same measurements were included for the pooled analysis [22]. Briefly, safety was analyzed in all randomized women who received at least 1 dose of study drug in the endometriosis patient population. Adverse events (AEs), clinical safety laboratory parameters (including lipid profiles), and BMD were analyzed. Adverse events were coded using the Medical Dictionary for Regulatory Activities (MedDRA), versions 18.0 (for Elaris EM-I) and 19.0 (for Elaris EM-II). The severity of each AE was rated by the investigator as mild, moderate, or severe. Serious AEs were defined as life-threatening, requiring hospitalization or medical or surgical intervention to prevent a serious outcome or events resulting in persistent disability or death. Treatment-emergent AEs were defined as AEs with a start date on or after the first dose of study drug and up to 30 days after the last dose of study drug.

### Statistical analyses

The primary analysis set was collected from EM-I and EM-II. The modified intent-to-treat population included women who received at least 1 dose of randomized, double-blind placebo or elagolix in the pivotal studies. Baseline data collected from the e-Diary were based on the last 35 days during the screening period prior to and including study day 1. A logistic regression model with treatment as the main effect and baseline value as a covariate was used to determine the *p* value for the difference in proportion of responders regarding dysmenorrhea and NMPP at month 3 between each elagolix treatment group and placebo. Sensitivity analyses were also conducted for the co-primary endpoints of responders regarding dysmenorrhea and NMPP at month 3. The primary analysis was repeated using a χ^2^ test along with a 2-sided 97.5% CI for the difference based on the normal approximation to the binomial distribution, with non-responders imputation (patients who prematurely discontinued the study drug at or before month 3), using mixed-imputation (patients who prematurely discontinued the study drug at or before month 3 due to AEs were considered non-responders), or with modification criteria for increased rescue analgesic use (patients were characterized as non-responders if they had a ≥ 15% increase in average pill count of NSAIDs and/or opioid analgesics).

Data from the modified intent-to-treat population were used to analyze the efficacy of elagolix across subgroups. For the co-primary endpoints, subgroup analysis utilized logistic regression with overall responder as the response variable, baseline pain value as a covariate, subgroup and treatment as main effects, and a treatment-by-subgroup interaction term. Subgroups were prespecified and nominal *p* values unadjusted for multiplicity are reported. For PGIC, subgroup analysis utilized logistic regression with subgroup and treatment as main effects, and a treatment-by-subgroup interaction term. For NRS, subgroup analysis utilized a mixed model repeated measure (MMRM) with baseline NRS score as a covariate; treatment, visit and subgroup as main effects; and treatment-by-subgroup, treatment-by-visit, subgroup-by-visit, treatment-by-subgroup-by-visit interaction terms. For EHP-30, subgroup analyses utilized analysis of covariance model with baseline EHP-30 as a covariate, treatment and subgroup as main effects, and a treatment-by-subgroup interaction term.

Statistical analyses were performed for the safety assessment of elagolix administered at both doses compared with placebo (elagolix dose groups were not compared against each other) to assess the effect of elagolix on AEs, laboratory values, vital signs, and BMD. Analyses were performed on all randomized women who received at least 1 dose of study drug within the first 6 months of placebo-controlled treatment. For the integrated safety analysis, AE data were summarized using MedDRA (version 19.01) system organ class and preferred terms. Analyses include treatment-emergent AEs defined as AEs with an onset date on or after the first dose of study drug and no more than 30 days after discontinuation of study drug. Fisher’s exact test was used to analyze comparisons between placebo and elagolix-treated groups for any event and for each preferred term. The between-group mean change from baseline with the 95% CI and SE was analyzed for laboratory variables. For BMD, the change from baseline was compared between each elagolix dose group and placebo using an analysis of covariance model with baseline value as a covariate and treatment group as the factor; the between-group mean change from baseline with the 95% CI, SE, and *p* value is reported. All *p* values reported are 2-sided.

Statistical analyses for this study were performed using SAS Version 9.3 or later (SAS Institute, Inc, Cary, NC) in a UNIX environment.

## Results

Pooled together, a total of 1686 women were treated in the EM-I and EM-II studies with 1285 (76.2%) completing the treatment period. Baseline characteristics were consistent among treatment groups [22]. Details regarding enrollment, follow-up rates, and reasons for trial discontinuation were also previously published [22].

### Co-primary endpoints

At month 3, the proportions of responders who met the co-primary endpoints in the pooled analysis of EM-I and EM-II data were significantly greater (*p* < 0.001) among women who received either dosage of elagolix vs. those who received placebo, which is consistent with the efficacy demonstrated in the individual studies [22]. At month 3, the percentage of women who had a clinically meaningful reduction in dysmenorrhea and experienced decreased or stable use of rescue analgesic agents was 45.0% in the elagolix 150-mg QD group, 74.2% in the elagolix 200-mg BID group, and 21.1% in the placebo group. Similar results were demonstrated in the percentage of women who had a clinically meaningful reduction in NMPP and decreased or stable use of rescue analgesic agents at month 3. For reductions in NMPP, 50.1% of women were responders in the elagolix 150-mg QD group, 56.1% were responders in the elagolix 200-mg BID group, and 36.5% were responders in the placebo group. In the pooled analysis at month 6, the efficacy of elagolix at both doses for dysmenorrhea and NMPP was persistent and clinically significant vs. placebo (dysmenorrhea: 44.0% with elagolix 150 mg QD, 76.1% with elagolix 200 mg BID, and 24.2% with placebo; NMPP: 48.5% with elagolix 150 mg QD, 62.2% with elagolix 200 mg BID, and 37.7% with placebo; *p* < 0.001 for all).

### Subgroup analyses for elagolix efficacy

**Fig. 1:**
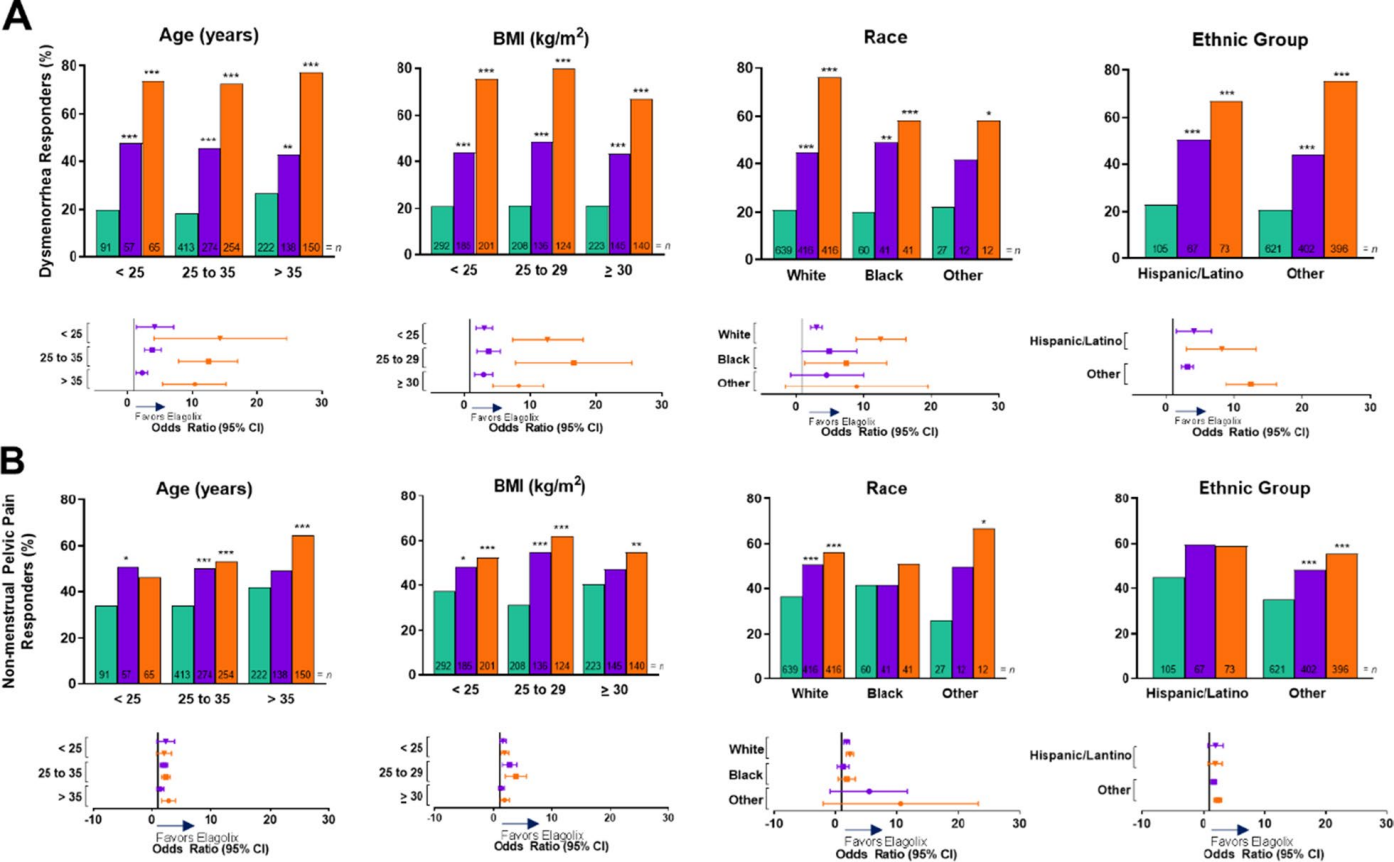
Co-primary endpoints at month 3 for integrated Elaris EM-I and Elaris EM-II by baseline demographic subgroups. **A** Dysmenorrhea responders. **B** Non-menstrual pelvic pain responders. Ratios equal the number of responders over the total number of women in each treatment group per subgroup. Green indicates placebo, purple indicates elagolix 150 mg QD and orange indicates elagolix 200 mg BID. *BID* twice daily; *BMI* body mass index; *QD* once daily

**Fig. 2:**
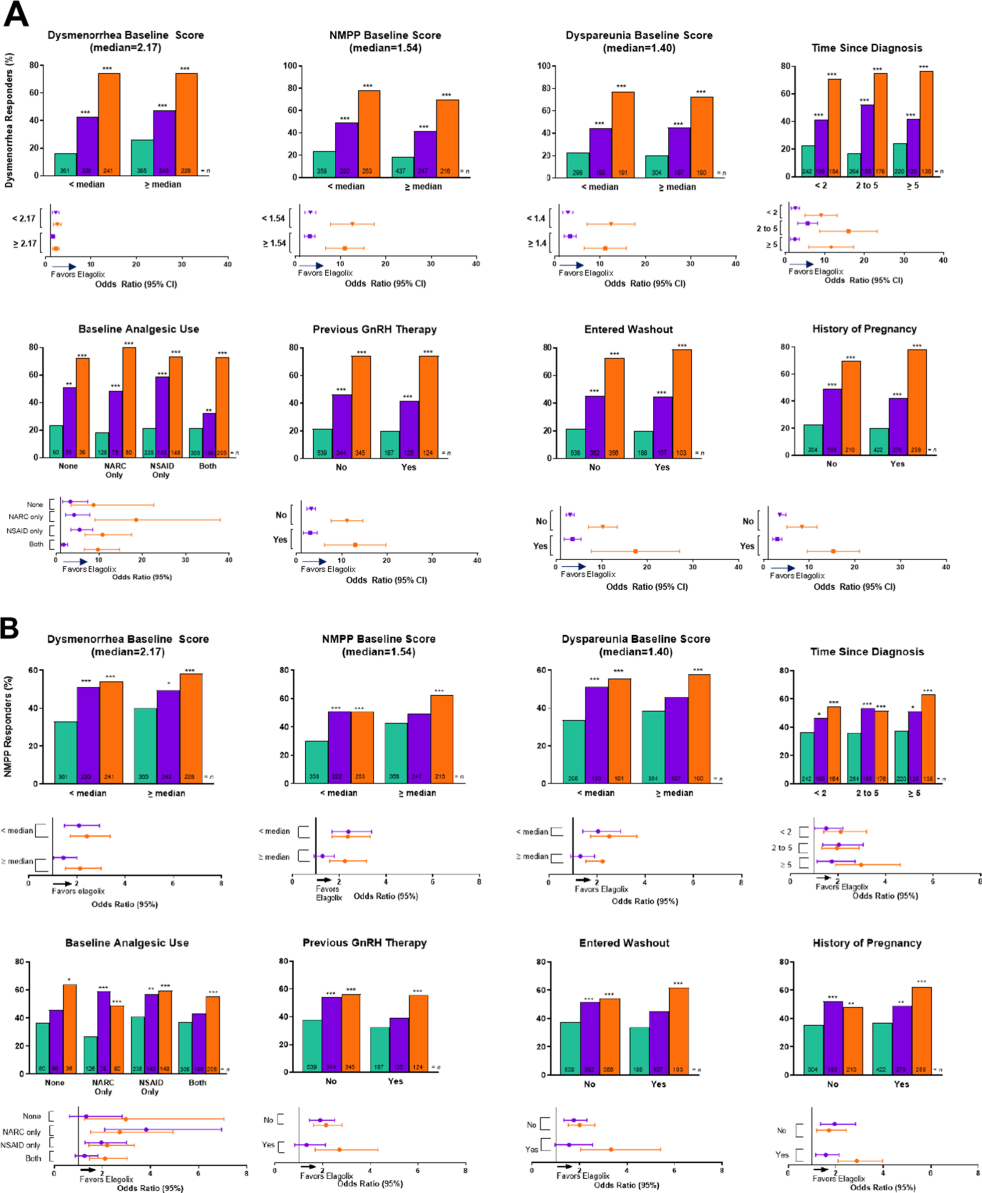
Co-primary endpoints at month 3 for integrated Elaris EM-I and Elaris EM-II by baseline disease severity subgroups. The treatment by subgroup interaction was significant (*p < 0.05, **p < 0.01). *p* values were obtained from a logistic regression model: responder/non-responder = base pain score + treatment + subgroup + treatment × subgroup for dysmenorrhea responders by previous analgesic use and history of pregnancy at baseline subgroups (**A**), and non-menstrual pelvic pain responders, non-menstrual pelvic pain baseline score, and history of pregnancy at baseline subgroups (**B**). Previous GnRH therapy includes both GnRH agonists and antagonists. Ratios equal the number of responders over the total number of women in each treatment group per subgroup. Green indicates placebo, purple indicates elagolix 150 mg QD, and orange indicates elagolix 200 mg BID. *BID* twice daily; *DYS* dysmenorrhea; *DYSP* dyspareunia; *GnRH* gonadotropin-releasing hormone; *NARC* narcotic/opioid; *NMPP* non-menstrual pelvic pain; *NSAID* non-steroidal anti-inflammatory drug; *QD* once daily

For each co-primary endpoint, baseline demographic and disease severity subgroups were investigated to assess potential differences in treatment effect of elagolix across subgroup levels from the pooled dataset. In general, at month 3, the number of women who met the co-primary endpoints with elagolix at 150 mg QD and 200 mg BID compared with placebo was independent of the analyzed subgroups including age, BMI, race, ethnic group, baseline dysmenorrhea score, baseline NMPP score, baseline dyspareunia score, time since diagnosis, baseline analgesic use, previous GnRH therapy, participation in the washout period (ie, baseline use of hormonal therapy), and history of pregnancy (Figs. 1 and 2).

Treatment by subgroup interaction was not statistically significant for any baseline demographic subgroups for dysmenorrhea or NMPP (Fig. 1A, B). However, treatment by subgroup interaction was statistically significant for baseline analgesic use (*p* < 0.01) and previous history of pregnancy (*p* < 0.05) for dysmenorrhea (Fig. 2A). Additionally, treatment by subgroup interaction was significant for baseline NMPP score (*p* < 0.05) and history of pregnancy (*p* < 0.05) for NMPP (Fig. 2B). These differences were not considered clinically important.

At month 3, for both elagolix dosages, the percentages of responders for dysmenorrhea across all subgroups were statistically greater compared with placebo, except for the “Other” race group taking elagolix 150 mg QD (Supplemental Figs. 1A and 2A). At month 3, for both elagolix dosages, the percentage of responders for NMPP followed the same trend for all subgroups; however, statistical significance between elagolix (both dosages) and placebo was not demonstrated for all subgroups (Supplemental Figs. 1B and 2B). Specifically, statistical significance was not found among NMPP responders receiving elagolix 200 mg BID, compared with placebo, for women aged younger than 25 years or in women who identified as Black or Hispanic/Latino, nor among NMPP responders taking elagolix 150 mg QD vs placebo in the following subgroups: age > 35 years, BMI ≥ 30 kg/m^2^, Black race, Other race, Hispanic/Latino ethnicity (Supplemental Fig. 1B), NMPP baseline score ≥ 1.54, dyspareunia baseline score ≥ 1.40, no baseline analgesic use, baseline analgesic use of both NSAIDs and opioids, entered washout after previous use of hormonal therapy, and previous GnRH therapy (Supplemental Fig. 2B).

### Subgroup analyses for health-related, quality-of-life measures

Results from the NRS, PGIC, and EHP-30 assessments were analyzed to evaluate the effect of elagolix on the women’s HRQoL across subgroups. Table 1 shows that for the NRS, participant-reported reduction in pain at month 3 was significant across all subgroups with elagolix 200 mg BID and most subgroups with elagolix 150 mg QD (Table 1).

**Table 1:** Numeric rating scale^a^ changes from baseline to month 3 for integrated Elaris EM-I and II

Parameter	Treatment	N	LS means (SE)	LS mean of difference (SE)	97.5% CI	Two-sided *p* value^b^
*Age*						
< 25 years						
	Placebo	75	− 1.13 (0.197)			
	150 mg QD	55	− 1.78 (0.242)	− 0.65 (0.312)	(− 1.35, 0.05)	0.037*
	200 mg BID	58	− 1.87 (0.232)	− 0.75 (0.304)	(− 1.43, − 0.06)	0.014*
25–35 years						
	Placebo	361	− 1.06 (0.092)			
	150 mg QD	248	− 1.83 (0.112)	− 0.77 (0.144)	(− 1.09, − 0.45)	< 0.001***
	200 mg BID	226	− 2.50 (0.117)	− 1.44 (0.149)	(− 1.77, − 1.10)	< 0.001***
> 35 years						
	Placebo	205	− 1.50 (0.123)			
	150 mg QD	127	− 1.80 (0.157)	− 0.30 (0.200)	(− 0.75, 0.15)	0.130
	200 mg BID	138	− 2.66 (0.151)	− 1.16 (0.194)	(− 1.59, − 0.72)	< 0.001***
*BMI (kg/m*^*2*^*)*						
< 25						
	Placebo	255	− 1.31 (0.109)			
	150 mg QD	164	− 1.72 (0.137)	− 0.40 (0.175)	(− 0.79, − 0.01)	0.022*
	200 mg BID	178	− 2.31 (0.132)	− 1.00 (0.171)	(− 1.38, − 0.61)	< 0.001***
≥ 25–29						
	Placebo	178	− 1.13 (0.130)			
	150 mg QD	130	− 1.93 (0.157)	− 0.80 (0.204)	(− 1.26, − 0.34)	< 0.001***
	200 mg BID	114	− 2.79 (0.166)	− 1.66 (0.211)	(− 2.13, − 1.18)	< 0.001***
≥ 30						
	Placebo	205	− 1.14 (0.124)			
	150 mg QD	134	− 1.84 (0.154)	− 0.70 (0.197)	(− 1.14, − 0.26)	< 0.001***
	200 mg BID	127	− 2.36 (0.157)	− 1.23 (0.200)	(− 1.68, − 0.78)	< 0.001***
*Race*						
White						
	Placebo	568	− 1.20 (0.074)			
	150 mg QD	380	− 1.78 (0.091)	− 0.58 (0.117)	(− 0.84, − 0.31)	< 0.001***
	200 mg BID	376	− 2.45 (0.091)	− 1.24 (0.117)	(− 1.51, − 0.98)	< 0.001***
Black						
	Placebo	52	− 1.28 (0.243)			
	150 mg QD	38	− 2.04 (0.289)	− 0.76 (0.377)	(− 1.61, 0.08)	0.043*
	200 mg BID	35	− 2.53 (0.294)	− 1.24 (0.381)	(− 2.10, − 0.39)	0.001***
Other						
	Placebo	21	− 1.13 (0.372)			
	150 mg QD	12	− 2.30 (0.524)	− 1.17 (0.643)	(− 2.61, 0.28)	0.070
	200 mg BID	11	− 2.85 (0.538)	− 1.72 (0.654)	(− 3.18, − 0.25)	0.009**
*Ethnicity*						
Hispanic/latino						
	Placebo	92	− 1.53 (0.181)			
	150 mg QD	64	− 2.04 (0.224)	− 0.51 (0.288)	(− 1.16, 0.14)	0.077
	200 mg BID	64	− 2.58 (0.220)	− 1.05 (0.285)	(− 1.69, − 0.41)	< 0.001***
Other						
	Placebo	549	− 1.15 (0.075)			
	150 mg QD	366	− 1.78 (0.092)	− 0.63 (0.119)	(− 0.89, − 0.36)	< 0.001***
	200 mg BID	358	− 2.44 (0.093)	− 1.29 (0.120)	(− 1.56, − 1.02)	< 0.001***
*Baseline dysmenorrhea*						
< Median = 2.17						
	Placebo	321	− 1.03 (0.099)			
	150 mg QD	209	− 1.67 (0.123)	− 0.63 (0.157)	(− 0.99, − 0.28)	< 0.001***
	200 mg BID	220	− 2.25 (0.121)	− 1.22 (0.154)	(− 1.56, − 0.87)	< 0.001***
≥ Median = 2.17						
	Placebo	320	− 1.38 (0.099)			
	150 mg QD	221	− 1.97 (0.121)	− 0.59 (0.154)	(− 0.93, − 0.24)	< 0.001***
	200 mg BID	202	− 2.70 (0.125)	− 1.31 (0.157)	(− 1.67, − 0.96)	< 0.001***
*Baseline NMPP*						
< Median = 1.54						
	Placebo	320	− 1.04 (0.103)			
	150 mg QD	208	− 1.73 (0.127)	− 0.68 (0.157)	(− 1.04, − 0.33)	< 0.001***
	200 mg BID	228	− 2.22 (0.122)	− 1.17 (0.152)	(− 1.51, − 0.83)	< 0.001***
≥ Median = 1.54						
	Placebo	321	− 1.37 (0.103)			
	150 mg QD	222	− 1.90 (0.124)	− 0.53 (0.153)	(− 0.88, − 0.19)	< 0.001***
	200 mg BID	194	− 2.75 (0.131)	− 1.38 (0.160)	(− 1.74, − 1.02)	< 0.001***
*Baseline dyspareunia*						
< Median = 1.40						
	Placebo	260	− 1.09 (0.109)			
	150 mg QD	174	− 1.72 (0.134)	− 0.63 (0.172)	(− 1.02, − 0.25)	< 0.001***
	200 mg BID	171	− 2.34 (0.135)	− 1.25 (0.172)	(− 1.64, − 0.87)	< 0.001***
≥ Median = 1.40						
	Placebo	266	− 1.30 (0.108)			
	150 mg QD	181	− 1.80 (0.133)	− 0.50 (0.169)	(− 0.88, − 0.12)	0.003**
	200 mg BID	169	− 2.64 (0.136)	− 1.33 (0.172)	(− 1.72, − 0.95)	< 0.001***
*Baseline analgesic use*						
None						
	Placebo	48	− 1.47 (0.244)			
	150 mg QD	55	− 1.48 (0.243)	− 0.01 (0.344)	(− 0.78, 0.77)	0.986
	200 mg BID	32	− 2.88 (0.310)	− 1.41 (0.394)	(− 2.29, − 0.53)	< 0.001***
Opioid only						
	Placebo	116	− 0.74 (0.162)			
	150 mg QD	77	− 1.86 (0.205)	− 1.12 (0.261)	(− 1.71, − 0.54)	< 0.001***
	200 mg BID	71	− 2.17 (0.208)	− 1.43 (0.264)	(− 2.02, − 0.83)	< 0.001***
NSAID only						
	Placebo	212	− 1.41 (0.120)			
	150 mg QD	127	− 2.24 (0.156)	− 0.83 (0.197)	(− 1.27, − 0.39)	< 0.001***
	200 mg BID	134	− 2.49 (0.152)	− 1.08 (0.194)	(− 1.52, − 0.65)	< 0.001***
Both						
	Placebo	265	− 1.19 (0.106)			
	150 mg QD	171	− 1.58 (0.133)	− 0.39 (0.170)	(− 0.77, − 0.01)	0.021*
	200 mg BID	185	− 2.49 (0.129)	− 1.30 (0.168)	(− 1.68, − 0.93)	< 0.001***
*Time since endometriosis diagnosis*						
< 2 years						
	Placebo	214	− 1.29 (0.119)			
	150 mg QD	174	− 1.66 (0.135)	− 0.37 (0.180)	(− 0.77, 0.04)	0.042*
	200 mg BID	138	− 2.52 (0.151)	− 1.23 (0.193)	(− 1.67, − 0.80)	< 0.001***
2–5 years						
	Placebo	235	− 1.16 (0.114)			
	150 mg QD	146	− 1.85 (0.148)	− 0.69 (0.187)	(− 1.11, − 0.27)	< 0.001***
	200 mg BID	155	− 2.35 (0.142)	− 1.19 (0.182)	(− 1.60, − 0.78)	< 0.001***
≥ 5 years						
	Placebo	192	− 1.17 (0.126)			
	150 mg QD	110	− 2.02 (0.168)	− 0.85 (0.210)	(− 1.32, − 0.38)	< 0.001***
	200 mg BID	128	− 2.54 (0.157)	− 1.37 (0.201)	(− 1.82, − 0.92)	< 0.001***
*Previous GnRH therapy*						
No						
	Placebo	476	− 1.27 (0.080)			
	150 mg QD	319	− 1.97 (0.099)	− 0.70 (0.127)	(− 0.99, − 0.41)	< 0.001***
	200 mg BID	309	− 2.49 (0.100)	− 1.22 (0.128)	(− 1.51, − 0.93)	< 0.001***
Yes						
	Placebo	165	− 1.03 (0.136)			
	150 mg QD	111	− 1.39 (0.167)	− 0.36 (0.215)	(− 0.84, 0.12)	0.095
	200 mg BID	113	− 2.41 (0.166)	− 1.38 (0.214)	(− 1.86, − 0.90)	< 0.001***
*Entered washout*						
No						
	Placebo	470	− 1.24 (0.080)			
	150 mg QD	328	− 1.86 (0.098)	− 0.62 (0.126)	(− 0.90, − 0.33)	< 0.001***
	200 mg BID	316	− 2.43 (0.099)	− 1.19 (0.128)	(− 1.47, − 0.90)	< 0.001***
Yes						
	Placebo	171	− 1.10 (0.135)			
	150 mg QD	102	− 1.66 (0.177)	− 0.56 (0.223)	(− 1.06, − 0.06)	0.013*
	200 mg BID	106	− 2.58 (0.173)	− 1.48 (0.220)	(− 1.97, − 0.99)	< 0.001***
*History of pregnancy*						
No						
	Placebo	278	− 1.28 (0.099)			
	150 mg QD	173	− 1.80 (0.126)	− 0.52 (0.160)	(− 0.88, − 0.16)	0.001***
	200 mg BID	184	− 2.21 (0.121)	− 0.94 (0.157)	(− 1.29, − 0.58)	< 0.001***
Yes						
	Placebo	363	− 1.15 (0.086)			
	150 mg QD	257	− 1.82 (0.105)	− 0.66 (0.135)	(− 0.97, − 0.36)	< 0.001***
	200 mg BID	238	− 2.68 (0.108)	− 1.53 (0.138)	(− 1.84, − 1.22)	< 0.001***

*BID* twice daily, *BMI* body mass index, *EM-I/II* Elaris endometriosis studies I and II, *GnRH* gonadotropin-releasing hormone, *LS* least squares, *NMPP* non-menstrual pelvic pain, *NSAID* non-steroidal anti-inflammatory drug, *QD* once daily

^a^Pain scale ranges from 0 (none) to 10 (worst pain ever)

^b^*p* value for test of difference between each elagolix dosage group and placebo is from a mixed-effects model using repeated measures with treatment as the main effect, visit number as the repeated measure, baseline value, visit, subgroup, treatment by visit, subgroup by treatment, subgroup by visit, and subgroup by treatment by visit as covariates

*p < 0.05, **p < 0.01, and ***p < 0.001

**Fig. 3:**
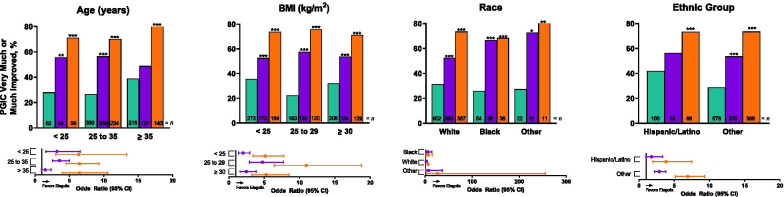
Patient Global Impression of Change (PGIC) at month 3 for integrated Elaris EM-I and Elaris EM-II by baseline demographic subgroups. PGIC uses a 7-point scale ranging from 1 = very much improved through 7 = very much worse. *p* values were obtained from a logistic regression model: “very much” or “much improved/otherwise” = treatment + subgroup + treatment × subgroup. *p* values for between-group comparisons were obtained using a contrast within the subgroup from the logistic regression model. *p < 0.05. Green indicates placebo, purple indicates elagolix 150 mg QD, and orange indicates elagolix 200 mg BID. *BID* twice daily; *BMI* body mass index; *QD* once daily

**Fig. 4:**
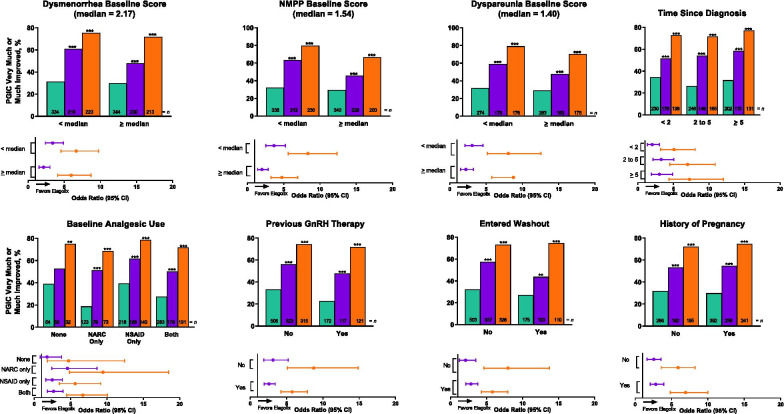
Patient Global Impression of Change (PGIC) at month 3 for integrated Elaris EM-I and Elaris EM-II by baseline disease severity subgroups. PGIC uses a 7-point scale ranging from 1 = very much improved through 7 = very much worse. *p* values were obtained from a logistic regression model: “very much” or “much improved/otherwise” = treatment + subgroup + treatment × subgroup. *p* values for between-group comparisons were obtained using a contrast within the subgroup from the logistic regression model. *p < 0.05. Green indicates placebo, purple indicates elagolix 150 mg QD, and orange indicates elagolix 200 mg BID. *BID* twice daily; *DYS* dysmenorrhea; *DYSP* dyspareunia; *GnRH* gonadotropin-releasing hormone; *NARC* narcotic/opioid; *NMPP* non-menstrual pelvic pain; *NSAID* non-steroidal anti-inflammatory drug; *QD* once daily

At month 3, PGIC scores indicated significantly improved endometriosis-related pain across all subgroups with elagolix 200 mg BID and most subgroups with elagolix 150 mg QD (Figs. 3 and 4 and Supplemental Figs. 3 and 4). Treatment by subgroup interaction was significant (*p* < 0.05) for age (Fig. 3) and NMPP score subgroups (Fig. 4).

Overall, women in the various subgroups experienced improvement in all domains of the EHP-30 at month 3. However, analysis of the EHP-30 results by subgroup showed several significant treatment-by-subgroup interactions (Supplemental Tables 1–6). For the pain domain, treatment-by-subgroup interactions were seen in the age, dysmenorrhea score, and NMPP score groups (*p* < 0.05; Supplemental Table 1). Significant interactions were also observed in ethnic and dysmenorrhea score groups for the control and powerlessness domain (*p* < 0.05; Supplemental Table 2). For the emotional well-being domain, interactions were seen in the NMPP score and previous GnRH analog treatment groups (*p* < 0.05; Supplemental Table 3). The social support domain revealed interactions for previous GnRH analog treatment (*p* < 0.05), dysmenorrhea score, NMPP score, and history of pregnancy groups (*p* < 0.01; Supplemental Table 4). For the self-image domain, no significant subgroup interactions were observed (Supplemental Table 5). For the sexual intercourse domain, interactions were seen in the group that used baseline analgesics (*p* < 0.05) and the groups that identified race and history of pregnancy (*p* < 0.01; Supplemental Table 6).

### Safety analysis

Adverse events for the Elaris EM-I and EM-II studies were previously reported by Taylor et al. [22]. In a subgroup analysis, AEs were described for the following categories: age, time since diagnosis, geographic region, and history of depression (Supplemental Table 7). Most AEs were classified as mild or moderate, which is consistent with the degrees of severity reported in previous studies [22].

## Discussion

As previously shown in the EM-I and EM-II phase 3 clinical trials, elagolix at 150 mg QD and 200 mg BID provides clinically meaningful reductions in dysmenorrhea and NMPP vs. placebo. Here, we pooled the data from both EM-I and EM-II and analyzed the co-primary efficacy endpoints for subgroups of patients with various baseline demographic and disease severity characteristics. Our results demonstrate that the effect of elagolix at both doses is consistent among all subgroups, with a greater proportion of responders for both dysmenorrhea and NMPP compared with placebo. There was no statistical comparison between the 2 elagolix dose groups; however, in the group receiving higher doses of elagolix, the proportion of responders vs. placebo appears to be greater than for the group receiving lower doses of elagolix, especially for dysmenorrhea. For NMPP, there were some instances where the responses were similar vs. placebo for either treatment dosage. Dysmenorrhea has been demonstrated to be mostly dependent on cyclic changes in ovarian hormones, whereas the mechanism of NMPP is more complex, suggesting why variations may have occurred in the NMPP response subgroups.

In this analysis, 2 disease severity subgroups demonstrate significant treatment by subgroup interaction. For dysmenorrhea, there is significant interaction in both the baseline analgesic use and history of pregnancy subgroups, and for NMPP, there is significant interaction in both the NMPP baseline score and history of pregnancy subgroups.

For women in the baseline analgesic use subgroup who were taking both NSAIDs and opioid analgesics at baseline, it appears that the proportion of women with a response in dysmenorrhea pain with the lower dose of elagolix was smaller than that of women who were not taking any analgesics at baseline or who were only taking NSAIDs alone or opioids alone. We postulate that women who are taking both NSAIDs and opioid analgesics at baseline have more severe pain; thus, a higher dose of elagolix is necessary to achieve a response in this subgroup of patients.

In NMPP responders in the baseline NMPP score subgroup, 2 circumstances are worth noting. In women with NMPP scores above the median, there appears to be a greater placebo effect than in women with NMPP scores below the median; therefore, the proportion of responders at the lower dosage of elagolix is not significant compared with placebo. Whereas, in women with NMPP scores below the median, the proportion of responders to elagolix at the lower dosage is significantly greater compared with placebo (*p* < 0.001; Fig. 2B). And, in women with NMPP scores below the median, it appears there is a similar proportion of responders to both the low and high dosages of elagolix, suggesting that when starting with a lower NMPP score, the lower dosage of elagolix is equally effective at reaching the co-primary endpoints as the higher dosage. These data are consistent with a recent study demonstrating a reduction in pain during bleeding and nonbleeding days after 6 months of treatment with both low and high dosages of elagolix [28].

Interestingly, in women who have both dysmenorrhea and NMPP, there was significant treatment by subgroup interaction in the history of pregnancy subgroup. It appears that at a higher dosage of elagolix, the magnitude of response seems to be greater for dysmenorrhea and NMPP in women with a previous history of pregnancy compared with women who have never been pregnant.

Many women with endometriosis experience a reduction in HRQoL [29, 30]. Specifically, pelvic pain associated with endometriosis has been shown to negatively affect HRQoL [31].

As measured on the NRS and PGIC scales, our findings demonstrate that elagolix 200 mg BID significantly improves endometriosis-related pain across all prespecified subgroups. The effect was less robust for the NRS and PGIC scales with elagolix 150 mg QD. Interestingly, for each of the NRS and PGIC scales, a few subgroups showed effects that were less than statistically significant; for both these measures, a lack of significant effect was seen for women who were older than 35 years, for women of Hispanic/Latino ethnicity, and for those who had reported no baseline analgesic use. We note that in women aged older than 35 years, a large placebo effect was observed for the NRS measurements (Table 1); this may provide an explanation for the lack of statistically significant effect seen in this age group. Overall, however, elagolix appears to significantly improve pain, and thus the HRQoL, for women across many demographically and clinically defined baseline characteristics.

Our findings are consistent with those of Pokrzywinski et al. [27] and Taylor et al. [32]. Pokrzywinski et al. [27] found that women with endometriosis in the EM-I and EM-2 trials who showed a clinical response to elagolix for dysmenorrhea or NMPP experienced improvements in HRQoL, as measured by the EHP-30. In an analysis of pooled data from the same 2 studies, Taylor et al. [32] found that women with moderate to severe endometriosis-associated pain who received elagolix showed clinically meaningful improvements in EHP-30 measurements of HRQoL. Neither of these previous studies used subgroup analyses to evaluate the effects of elagolix on the HRQoL of women with different baseline characteristics. In our post hoc analysis, results on the EHP-30 scale demonstrate that elagolix improves HRQoL in 6 different domains (pain, control and powerlessness, emotional well-being, social support, self-image, and sexual relationships) across prespecified subgroups.

The same limitations of the individual clinical trials applied to the pooled analysis. Limitations existed within the entry criteria and length of the intervention period, and generalizability of the results. The effect of elagolix was not examined in women with a *z* score of less than − 1.5 for BMD or in women with large endometriomas. Also, staging of endometriosis was incomplete and not used in the analysis as surgical diagnoses had occurred within the previous 10 years. Therefore, we cannot discuss the relative impact of treatment based on extent of disease, except in relation to the extent of symptoms, which have been shown to correlate poorly with disease stage [33]. To date, head-to-head safety comparisons between elagolix and other GnRH analogs, including relugolix, cetrorelix, and ganirelix, have not been published. Medical therapy may be sufficient to reduce symptoms and signs of endometriosis for most patients; however, deep infiltrating and extrapelvic endometriosis are especially challenging to treat [34–36]. In a large number of these patients, eradication of endometriosis-associated adhesions/fibrosis may be necessary to restore normal pelvic anatomy and function, which requires a nerve-sparing and vascular-sparing approach [36, 37]. Further, definitive conclusions cannot be made based on post hoc analyses.

In general, despite the variations seen within the demographic and disease severity subgroups, the trends were similar. Findings from the subgroup analyses demonstrate that age, BMI, race, ethnicity, baseline dysmenorrhea scores, baseline NMPP scores, baseline dyspareunia scores, time since diagnosis, analgesic use at baseline, previous GnRH analog therapy (including the use of both agonists and antagonists), participation in the washout period, and history of pregnancy, had little effect on the efficacy of elagolix. Additionally, our findings demonstrate that elagolix treatment was generally effective in improving the HRQoL of women across these prespecified subgroups. Given these findings, it may be prudent for physicians to consider elagolix to treat their patients who have unresolved endometriosis pain and who may present with a variety of demographic and clinical characteristics. These patients may include women who present with various levels of pain related to dysmenorrhea, NMPP, and/or dyspareunia, or those with different treatment histories (eg, previous use of analgesics [opioids, NSAIDs, both, or none], previous GnRH analog therapy, or previous hormonal therapy). Overall, this study may aid physicians in identifying elagolix as an effective treatment for a variety of women with endometriosis, despite variations in their demographics or medical histories.

## Conclusions

In this study, we have identified that elagolix is effective in reducing dysmenorrhea and NMPP, as well as improving HRQoL, compared with placebo across numerous subgroups of women with various baseline characteristics, encompassing demographic categories as well as a wide range of clinical variables that characterize patients with endometriosis.

## Supplementary Information

**Additional file 1: Supplemental Table 1.** Endometriosis Health Profile-30 at month 3 – pain; **Supplemental Table 2**. Endometriosis Health Profile-30 at month 3 – control and powerlessness; **Supplemental Table 3**. Endometriosis Health Profile-30 at month 3 – emotional well-being; **Supplemental Table 4**. Endometriosis Health Profile-30 at month 3 – social support; **Supplemental Table 5**. Endometriosis Health Profile-30 at Month 3 – self-image; **Supplemental Table 6**. Endometriosis Health Profile-30 at month 3 – sexual intercourse; **Supplemental Table 7**. Adverse events from integrated Elaris EM-I and Elaris EM-II by selected subgroup; **Supplemental Figure 1**. Co-primary endpoints at month 3 for integrated Elaris EM-I and II by baseline demographic subgroups; **Supplemental Figure 2**. Co-primary endpoints at month 3 for integrated Elaris EM-I and Elaris EM-II by disease severity subgroups; **Supplemental Figure 3**. Patient Global Impression of Change (PGIC) at month 3 for integrated Elaris EM-I and Elaris EM-II by baseline demographic subgroups; **Supplemental Figure 4**. Patient Global Impression of Change (PGIC) at month 3 for integrated Elaris EM-I and Elaris EM-II by disease severity subgroups.

## Data Availability

AbbVie, Inc., is committed to responsible data sharing regarding the clinical trials we sponsor. This includes access to anonymized, individual and trial-level data (analysis data sets), as well as other information (eg, protocols and Clinical Study Reports), as long as the trials are not part of an ongoing or planned regulatory submission. This includes requests for clinical trial data for unlicensed products and indications. These clinical trial data can be requested by any qualified researchers who engage in rigorous, independent scientific research, and will be provided following review and approval of a research proposal and Statistical Analysis Plan (SAP) and execution of a Data Sharing Agreement (DSA). Data requests can be submitted at any time and the data will be accessible for 12 months, with possible extensions considered. For more information on the process, or to submit a request, visit the following link: https://www.abbvie.com/our-science/clinical-trials/clinical-trials-data-and-information-sharing/data-and-information-sharing-with-qualified-researchers.html.

## Abbreviations

AE: Adverse event
BID: Twice a day
BMI: Body mass index
BMD: Bone mineral density
EHP-30: Endometriosis Health Profile-30
EM-I: Elaris endometriosis study I
EM-II: Elaris endometriosis study II
GnRH: Gonadotropin-releasing hormone
HRQoL: Health-related quality of life
NMPP: Non-menstrual pelvic pain
NSAID: Non-steroidal anti-inflammatory drug
NRS: Numeric rating scale
PGIC: Patient global impression of change
QD: Once a day
