# Supplementary material for: Reductions in endometriosis-associated pain among women treated with elagolix are consistent across a range of baseline characteristics reflective of real-world patients
Source: BMC Womens Health. 2021 Jun 16;21:246. doi: 10.1186/s12905-021-01385-3 (PMC8210385; doi:10.1186/s12905-021-01385-3)

Reductions in endometriosis-associated pain among women treated with elagolix are consistent across a range of baseline characteristics reflective of real-world patients

Mauricio S. Abrao[1,2], Eric Surrey[3], Keith Gordon[4], Michael C. Snabes[4], Hui Wang[4], Horia Ijacu[4], and Hugh S. Taylor[5]

[1]Endometriosis Section, Gynecologic Division. Hospital das Clinicas HCFMUSP, Faculdade de Medicina, Universidade de Sao Paulo, São Paulo, São Paulo, Brazil

[2]Gynecologic Division, Hospital BP-A Beneficencia Portuguesa de Sao Paulo, São Paulo, São Paulo, Brazil

[3]Colorado Center for Reproductive Medicine, Lone Tree, CO, USA

[4]Departments of Clinical Development, Medical Affairs, and Statistics, AbbVie Inc., North Chicago, IL, USA

[5]Department of Obstetrics, Gynecology and Reproductive Sciences, Yale School of Medicine, New Haven, CT, USA

SUPPLEMENTAL MATERIALS

SUPPLEMENTAL TABLES

Supplemental Table 1. Endometriosis Health Profile-30 at month 3 – pain

Variable	*N*	Least squares means (SE)	Difference of least squares means (SE)	95% CI	*p* value[b]	Treatment by subgroup *p* value[c]	Subgroup *p* value[c]
Pain[a]							
Age group (< 25 years, 2 to 35 years, > 35 years)						0.036*	0.069
Placebo	616	−18.47 (0.958)					
Elagolix 150 mg QD	420	−25.84 (1.134)	−7.37 (1.48)	(−10.28, −4.46)	<0.001***		
Elagolix 200 mg BID	397	−35.59 (1.139)	−17.12 (1.49)	(−20.04, −14.20)	<0.001***		
BMI group (normal = < 25 kg/m^2, overweight = 25 to ≤ 29.9 kg/m^2, obese = ≥ 30 kg/m^2)						0.140	0.228
Placebo	614	−18.28 (0.790)					
Elagolix 150 mg QD	419	−26.81 (0.948)	−8.53 (1.23)	(−10.95, −6.11)	<0.001***		
Elagolix 200 mg BID	394	−35.69 (0.991)	−17.42 (1.27)	(−19.90, −14.93)	<0.001***		
Race group (White, Black, other)						0.236	0.958
Placebo	616	−16.26 (1.711)					
Elagolix 150 mg QD	420	−27.90 (2.162)	−11.64 (2.76)	(−17.05, −6.24)	<0.001***		
Elagolix 200 mg BID	397	−35.75 (2.303)	−19.49 (2.86)	(−25.11, −13.87)	<0.001***		
Ethnic group (Hispanic or Latino, other)						0.190	0.004**
Placebo	616	−17.85 (1.096)					
Elagolix 150 mg QD	420	−24.02 (1.327)	−6.17 (1.72)	(−9.54, −2.79)	<0.001***		
Elagolix 200 mg BID	397	−34.65 (1.342)	−16.80 (1.73)	(−20.20, −13.40)	<0.001***		
Baseline dysmenorrhea (< median = 2.17, ≥ median = 2.17)						0.047*	<0.001***
Placebo	616	−18.49 (0.774)					
Elagolix 150 mg QD	420	−26.87 (0.937)	−8.39 (1.22)	(−10.77, −6.00)	<0.001***		
Elagolix 200 mg BID	397	−35.57 (0.965)	−17.08 (1.24)	(−19.51, −14.66)	<0.001***		
Baseline NMPP (< median = 1.54, ≥ median = 1.54)						0.011*	<0.001***
Placebo	616	−18.46 (0.767)					

Elagolix 150 mg QD	420	−27.02 (0.929)	−8.56 (1.20)	(−10.92, −6.20)	<0.001***		
Elagolix 200 mg BID	397	−35.28 (0.958)	−16.81 (1.23)	(−19.22, −14.40)	<0.001***		
Baseline dyspareunia (< median = 1.40, ≥ median = 1.40)						0.676	0.004**
Placebo	504	−18.83 (0.860)					
Elagolix 150 mg QD	349	−27.10 (1.034)	−8.27 (1.35)	(−10.91, −5.63)	<0.001***		
Elagolix 200 mg BID	320	−36.67 (1.080)	−17.84 (1.38)	(−20.55, −15.14)	<0.001***		
Time since endometriosis diagnosis (< 2 years, 2 to <5 years, ≥ 5 years)						0.883	0.035*
Placebo	616	−18.54 (0.782)					
Elagolix 150 mg QD	420	−27.07 (0.958)	−8.53 (1.24)	(−10.95, −6.10)	<0.001***		
Elagolix 200 mg BID	396	−35.54 (0.976)	−16.99 (1.25)	(−19.45, −14.54)	<0.001***		
Baseline analgesic use (none, opioids, NSAID, both)						0.065	<0.001***
Placebo	616	−18.13 (0.950)					
Elagolix 150 mg QD	420	−26.50 (1.026)	−8.37 (1.40)	(−11.12, −5.63)	<0.001***		
Elagolix 200 mg BID	397	−36.48 (1.190)	−18.35 (1.52)	(−21.33, −15.36)	<0.001***		
Previous GnRH therapy (yes, no)						0.529	0.089
Placebo	616	−17.68 (0.898)					
Elagolix 150 mg QD	420	−26.16 (1.073)	−8.47 (1.40)	(−11.22, −5.73)	<0.001***		
Elagolix 200 mg BID	397	−35.56 (1.093)	−17.88 (1.41)	(−20.65, −15.10)	<0.001***		
Entered washout (yes, no)						0.527	0.677
Placebo	616	−18.20 (0.884)					
Elagolix 150 mg QD	420	−26.33 (1.116)	−8.13 (1.42)	(−10.92, −5.34)	<0.001***		
Elagolix 200 mg BID	397	−35.96 (1.119)	−17.77 (1.43)	(−20.57, −14.97)	<0.001***		
History of pregnancy at baseline (yes, no)						0.504	0.783
Placebo	616	−18.61 (0.789)					
Elagolix 150 mg QD	420	−26.87 (0.966)	−8.27 (1.25)	(−10.71, −5.82)	<0.001***		
Elagolix 200 mg BID	397	−35.50 (0.981)	−16.89 (1.26)	(−19.36, −14.42)	<0.001***		

[a]Each dimension for the core and modular questionnaire is calculated on a scale from 0 = best possible health status to 100 = worst possible health status.

[b]*p* value for test of difference between each elagolix dose group and placebo at each post-baseline time point is from an ANCOVA model with treatment as the main effect, baseline value, subgroup and treatment × subgroup as covariates.

[c]*p* values are obtained from an ANCOVA model: change from baseline = treatment + baseline value + subgroup + treatment × subgroup.

*$p<0.05$. **$p<0.01$. ***$p<0.001$.

ANCOVA analysis of covariance; *BID* twice daily; *BMI* body mass index; *GnRH* gonadotropin-releasing hormone; *NMPP* non-menstrual pelvic pain; *NSAID* non-steroidal anti-inflammatory drug; *QD* once daily.

Supplemental Table 2. Endometriosis Health Profile-30 at month 3 – control and powerlessness

Variable	*N*	Least squares means (SE)	Difference of least squares means (SE)	95% CI	*p* value[b]	Treatment by subgroup *p* value[c]	Subgroup *p* value[c]
**Control and Powerlessness[a]**							
Age group (< 25 years, 25 to 35 years, > 35 years)						0.353	0.640
Placebo	626	−24.92 (1.141)					
Elagolix 150 mg QD	420	−32.06 (1.379)	−7.14 (1.79)	(−10.65, −3.63)	<0.001***		
Elagolix 200 mg BID	401	−42.83 (1.362)	−17.91 (1.78)	(−21.40, −14.43)	<0.001***		
BMI group (mormal = < 25 kg/m^2, overweight = 25 to ≤ 29.9 kg/m^2, obese = ≥ 30 kg/m^2)						0.300	0.399
Placebo	624	−24.76 (0.946)					
Elagolix 150 mg QD	419	−32.84 (1.145)	−8.08 (1.49)	(−11.00, −5.17)	<0.001***		
Elagolix 200 mg BID	398	−42.42 (1.190)	−17.66 (1.52)	(−20.64, −14.68)	<0.001***		
Race group (White, Black, other)						0.241	0.908
Placebo	626	−22.20 (2.048)					
Elagolix 150 mg QD	420	−35.32 (2.606)	−13.12 (3.31)	(−19.62, −6.62)	<0.001***		
Elagolix 200 mg BID	401	−43.66 (2.776)	−21.46 (3.45)	(−28.22, −14.70)	<0.001***		
Ethnic group (Hispanic or Latino, other)						0.025*	0.067
Placebo	626	−25.51 (1.308)					
Elagolix 150 mg QD	420	−29.54 (1.589)	−4.03 (2.06)	(−8.07, 0.01)	0.050*		
Elagolix 200 mg BID	401	−41.55 (1.618)	−16.04 (2.08)	(−20.12, −11.96)	<0.001***		
Baseline dysmenorrhea (< median = 2.17, ≥ median = 2.17)						0.040*	<0.001***
Placebo	626	−24.87 (0.926)					
Elagolix 150 mg QD	420	−32.88 (1.131)	−8.02 (1.46)	(−10.88, −5.15)	<0.001***		
Elagolix 200 mg BID	401	−42.25 (1.158)	−17.38 (1.48)	(−20.29, −14.47)	<0.001***		
Baseline NMPP (< median = 1.54, ≥ median = 1.54)						0.126	<0.001***
Placebo	626	−24.86 (0.925)					
Elagolix 150 mg QD	420	−32.97 (1.130)	−8.12 (1.46)	(−10.98, −5.25)	<0.001***		
Elagolix 200 mg BID	401	−42.10 (1.159)	−17.24 (1.48)	(−20.15, −14.33)	<0.001***		
Baseline dyspareunia (< median = 1.40, ≥ median = 1.40)						0.455	0.113
Placebo	511	−25.45 (1.033)					

Elagolix 150 mg QD	348	−33.08 (1.252)	−7.63 (1.62)	(−10.82, −4.45)	<0.001***		
Elagolix 200 mg BID	325	−43.69 (1.295)	−18.24 (1.66)	(−21.49, −14.99)	<0.001***		
Time since endometriosis diagnosis (< 2 years, 2 to < 5 years, ≥ 5 years)						0.536	0.078
Placebo	626	−24.91 (0.934)					
Elagolix 150 mg QD	420	−33.28 (1.154)	−8.37 (1.49)	(−11.28, −5.46)	<0.001***		
Elagolix 200 mg BID	400	−42.23 (1.170)	−17.32 (1.50)	(−20.26, −14.38)	<0.001***		
Baseline analgesic use (none, opioids, NSAID, both)						0.124	0.003**
Placebo	626	−24.96 (1.138)					
Elagolix 150 mg QD	420	−32.42 (1.249)	−7.46 (1.69)	(−10.78, −4.14)	<0.001***		
Elagolix 200 mg BID	401	−43.57 (1.439)	−18.61 (1.83)	(−22.21, −15.02)	<0.001***		
Previous GnRH therapy (yes, no)						0.086	0.612
Placebo	626	−23.86 (1.070)					
Elagolix 150 mg QD	420	−32.44 (1.298)	−8.57 (1.68)	(−11.87, −5.28)	<0.001***		
Elagolix 200 mg BID	401	−43.07 (1.311)	−19.21 (1.69)	(−22.52, −15.89)	<0.001***		
Entered washout (yes, no)						0.597	0.430
Placebo	626	−24.65 (1.054)					
Elagolix 150 mg QD	420	−31.96 (1.346)	−7.31 (1.71)	(−10.67, −3.96)	<0.001***		
Elagolix 200 mg BID	401	−42.44 (1.342)	−17.79 (1.71)	(−21.14, −14.44)	<0.001***		
History of pregnancy at baseline (yes, no)						0.135	0.140
Placebo	626	−24.90 (0.940)					
Elagolix 150 mg QD	420	−32.72 (1.161)	−7.82 (1.49)	(−10.75, −4.89)	<0.001***		
Elagolix 200 mg BID	401	−41.90 (1.175)	−16.99 1.50)	(−19.95, −14.04)	<0.001***		

[a]Each dimension for the core and modular questionnaire is calculated on a scale from 0 = best possible health status to 100 = worst possible health status.

[b]*p* value for test of difference between each elagolix dose group and placebo at each post-baseline time point is from an ANCOVA model with treatment as the main effect, baseline value, subgroup, and treatment × subgroup as covariates.

[c]*p* values are obtained from an ANCOVA model: change from baseline = treatment + baseline value + subgroup + treatment × subgroup.

*p<0.05. **p<0.01. ***p<0.001.

ANCOVA analysis of covariance; *BID* twice daily; *BMI* body mass index; *GnRH* gonadotropin-releasing hormone; *NMPP* non-menstrual pelvic pain; *NSAID* non-steroidal anti-inflammatory drug; *QD* once daily.

Supplemental Table 3. Endometriosis Health Profile-30 at month 3 – emotional well-being

Variable	*N*	Least squares means (SE)	Difference of least squares means (SE)	95% CI	*p* value[b]	Treatment by subgroup *p* value[c]	Subgroup *p* value[c]
**Emotional Well-being[a]**							
Age group (< 25 years, 25 to 35 years, > 35 years)						0.502	0.221
Placebo	627	−13.43 (0.920)					
Elagolix 150 mg QD	418	−17.30 (1.107)	−3.87 (1.44)	(−6.69, −1.05)	0.007**		
Elagolix 200 mg BID	396	−20.76 (1.118)	−7.33 (1.45)	(−10.17, −4.49)	<0.001***		
BMI group (normal = < 25 kg/m^2, overweight = 25 to ≤ 29.9 kg/m^2, obese = ≥ 30 kg/m^2)						0.361	0.936
Placebo	625	−13.59 (0.761)					
Elagolix 150 mg QD	417	−17.81 (0.925)	−4.22 (1.20)	(−6.57, −1.87)	<0.001***		
Elagolix 200 mg BID	393	−21.26 (0.964)	−7.66 (1.23)	(−10.07, −5.25)	<0.001***		
Race group (White, Black, other)						0.324	0.708
Placebo	627	−12.75 (1.681)					
Elagolix 150 mg QD	418	−18.92 (2.103)	−6.18 (2.69)	(−11.46, −0.90)	0.022*		
Elagolix 200 mg BID	396	−22.13 (2.232)	−9.38 (2.79)	(−14.86, −3.90)	<0.001***		
Ethnic group (Hispanic or Latino, other)						0.542	0.099
Placebo	627	−13.21 (1.052)					
Elagolix 150 mg QD	418	−16.15 (1.286)	−2.94 (1.66)	(−6.20, 0.32)	0.077		
Elagolix 200 mg BID	396	−20.79 (1.29)	−7.58 (1.67)	(−10.85, −4.31)	<0.001***		
Baseline dysmenorrhea (< median = 2.17, ≥ median = 2.17)						0.436	<0.001***
Placebo	627	−13.69 (0.749)					
Elagolix 150 mg QD	418	−17.81 (0.917)	−4.12 (1.18)	(−6.44, −1.80)	<0.001***		
Elagolix 200 mg BID	396	−21.10 (0.944)	−7.41 (1.20)	(−9.77, −5.04)	<0.001***		
Baseline NMPP (< median = 1.54, ≥ median = 1.54)						0.043*	<0.001***
Placebo	627	−13.67 (0.748)					
Elagolix 150 mg QD	418	−17.88 (0.917)	−4.21 (1.18)	(−6.53, −1.89)	<0.001***		
Elagolix 200 mg BID	396	−21.08 (0.944)	−7.40 (1.20)	(−9.77, −5.04)	<0.001***		
Baseline dyspareunia (< median = 1.40, ≥ median = 1.40)						0.286	0.029*
Placebo	511	−14.14 (0.841)					
Elagolix 150 mg QD	347	−18.02 (1.020)	−3.89 (1.32)	(−6.48, −1.29)	0.003**		

Elagolix 200 mg BID	319	−21.87 (1.064)	−7.73 (1.36)	(−10.39, −5.07)	<0.001***		
Time since endometriosis diagnosis (< 2 years, 2 to < 5 years, ≥ 5 years)						0.782	0.086
Placebo	627	−13.67 (0.754)					
Elagolix 150 mg QD	418	−17.97 (0.935)	−4.30 (1.20)	(−6.66, −1.95)	<0.001***		
Elagolix 200 mg BID	395	−21.05 (0.951)	−7.39 (1.21)	(−9.77, −5.01)	<0.001***		
Baseline analgesic use (none, opioid, NSAID, both)						0.607	0.008**
Placebo	627	−13.81 (0.921)					
Elagolix 150 mg QD	418	−17.61 (1.008)	−3.80 (1.37)	(−6.47, −1.12)	0.005**		
Elagolix 200 mg BID	396	−21.77 (1.159)	−7.96 (1.48)	(−10.86, −5.06)	<0.001***		
Previous GnRH therapy (yes, no)						0.045*	0.262
Placebo	627	−12.56 (0.862)					
Elagolix 150 mg QD	418	−17.39 (1.043)	−4.83 (1.35)	(−7.49, −2.18)	<0.001***		
Elagolix 200 mg BID	396	−21.65 (1.053)	−9.09 (1.36)	(−11.76, −6.42)	<0.001***		
Entered washout (yes, no)						0.522	0.552
Placebo	627	−13.89 (0.852)					
Elagolix 150 mg QD	418	−17.45 (1.087)	−3.56 (1.38)	(−6.27, −0.85)	0.010**		
Elagolix 200 mg BID	396	−21.74 (1.096)	−7.86 (1.39)	(−10.58, −5.13)	<0.001***		
History of pregnancy at baseline (yes, no)						0.179	0.043*
Placebo	627	−13.51 (0.758)					
Elagolix 150 mg QD	418	−17.80 (0.939)	−4.29 (1.21)	(−6.66, −1.92)	<0.001***		
Elagolix 200 mg BID	396	−20.85 (0.953)	−7.34 (1.22)	(−9.73, −4.95)	<0.001***		

[a]Each dimension for the core and modular questionnaire is calculated on a scale from 0 = best possible health status to 100 = worst possible health status.

[b]*p* value for test of difference between each elagolix dose group and placebo at each postbaseline time point is from an ANCOVA model with treatment as the main effect, baseline value, subgroup, and treatment × subgroup as covariates.

[c]*p* values are obtained from an ANCOVA model: change from baseline = treatment + baseline value + subgroup + treatment × subgroup.

$^{*}p<0.05$. $^{**}p<0.01$. $^{***}p<0.001$.

ANCOVA analysis of covariance; *BID* twice daily; *BMI* body mass index; *GnRH* gonadotropin-releasing hormone; *NMPP* non-menstrual pelvic pain; *NSAID* non-steroidal anti-inflammatory drug; *QD* once daily.

Supplemental Table 4. Endometriosis Health Profile-30 at month 3 – social support

Variable	*N*	Least squares means (SE)	Difference of least squares means (SE)	95% CI	*p* value[b]	Treatment by subgroup *p* value[c]	Subgroup *p* value[c]
**Social Support[a]**							
Age group (< 25 years, 25 to 35 years, > 35 years)						0.100	0.013*
Placebo	629	−12.25 (1.143)					
Elagolix 150 mg QD	422	−16.62 (1.376)	−4.37 (1.79)	(−7.88, −0.86)	0.015*		
Elagolix 200 mg BID	406	−23.73 (1.358)	−11.48 (1.78)	(−14.96, −8.00)	<0.001***		
BMI group (normal = < 25 kg/m^2, overweight = 25 to ≤ 29.9 kg/m^2, obese = ≥ 30 kg/m^2)						0.766	0.619
Placebo	627	−12.63 (0.947)					
Elagolix 150 mg QD	421	−17.96 (1.147)	−5.33 (1.49)	(−8.25, −2.41)	<0.001***		
Elagolix 200 mg BID	403	−24.11 (1.191)	−11.48 (1.52)	(−14.46, −8.49)	<0.001***		
Race group (White, Black, other)						0.294	0.978
Placebo	629	−11.73 (2.056)					
Elagolix 150 mg QD	422	−20.20 (2.616)	−8.47 (3.33)	(−14.99, −1.94)	0.011*		
Elagolix 200 mg BID	406	−23.18 (2.774)	−11.44 (3.45)	(−18.21, −4.67)	<0.001***		
Ethnic group (Hispanic or Latino, other)						0.085	0.030*
Placebo	629	−12.23 (1.312)					
Elagolix 150 mg QD	422	−14.72 (1.595)	−2.49 (2.07)	(−6.54, 1.56)	0.229		
Elagolix 200 mg BID	406	−23.77 (1.600)	−11.54 (2.07)	(−15.60, −7.48)	<0.001***		
Baseline dysmenorrhea (< median = 2.17, ≥ median = 2.17)						0.002**	<0.001***
Placebo	629	−12.57 (0.926)					
Elagolix 150 mg QD	422	−18.13 (1.130)	−5.56 (1.46)	(−8.43, −2.70)	<0.001***		
Elagolix 200 mg BID	406	−24.11 (1.153)	−11.54 (1.48)	(−14.45, −8.64)	<0.001***		
Baseline NMPP (< median = 1.54, ≥ median = 1.54)						0.010**	<0.001***
Placebo	629	−12.55 (0.923)					
Elagolix 150 mg QD	422	−18.26 (1.127)	−5.71 (1.46)	(−8.57, −2.85)	<0.001***		
Elagolix 200 mg BID	406	−23.98 (1.153)	−11.43 (1.48)	(−14.33, −8.54)	<0.001***		
Baseline dyspareunia (< median = 1.40, ≥ median = 1.40)						0.273	0.006**
Placebo	515	−12.80 (1.037)					
Elagolix 150 mg QD	350	−17.62 (1.258)	−4.81 (1.63)	(−8.01, −1.62)	0.003**		

Elagolix 200 mg BID	327	−25.09 (1.302)	−12.29 (1.67)	(−15.56, −9.02)	<0.001***		
Time since endometriosis diagnosis (< 2 years, 2 to < 5 years, ≥ 5 years)						0.473	<0.001***
Placebo	629	−12.55 (0.931)					
Elagolix 150 mg QD	422	−18.53 (1.152)	−5.98 (1.48)	(−8.89, −3.08)	<0.001***		
Elagolix 200 mg BID	405	−23.94 (1.162)	−11.40 (1.49)	(−14.32, −8.47)	<0.001***		
Baseline analgesic use (none, opioids, NSAID, both)						0.219	0.012*
Placebo	629	−12.79 (1.142)					
Elagolix 150 mg QD	422	−17.57 (1.249)	−4.78 (1.69)	(−8.10, −1.46)	0.005**		
Elagolix 200 mg BID	406	−24.84 (1.429)	−12.05 (1.83)	(−15.64, −8.47)	<0.001***		
Previous GnRH therapy (yes, no)						0.021*	0.145
Placebo	629	−10.86 (1.068)					
Elagolix 150 mg QD	422	−17.57 (1.290)	−6.71 (1.67)	(−9.99, −3.42)	<0.001***		
Elagolix 200 mg BID	406	−24.69 (1.304)	−13.83 (1.69)	(−17.13, −10.52)	<0.001***		
Entered washout (yes, no)						0.847	0.481
Placebo	629	−12.83 (1.061)					
Elagolix 150 mg QD	422	−17.96 (1.345)	−5.13 (1.71)	(−8.49, −1.77)	0.003**		
Elagolix 200 mg BID	406	−24.65 (1.345)	−11.81 (1.71)	(−15.18, −8.45)	<0.001***		
History of pregnancy at baseline (yes, no)						0.005**	0.109
Placebo	629	−12.58 (0.941)					
Elagolix 150 mg QD	422	−18.14 (1.160)	−5.55 (1.49)	(−8.48, −2.62)	<0.001***		
Elagolix 200 mg BID	406	−23.61 (1.168)	−11.03 (1.50)	(−13.97, −8.09)	<0.001***		

[a]Each dimension for the core and modular questionnaire is calculated on a scale from 0 = best possible health status to 100 = worst possible health status.

[b]*p* value for test of difference between each elagolix dose group and placebo at each post-baseline time point is from an ANCOVA model with treatment as the main effect, baseline value, subgroup, and treatment × subgroup as covariates.

[c]*p* values are obtained from an ANCOVA model: change from baseline = treatment + baseline value + subgroup + treatment × subgroup.

$^*p<0.05$. $^{**}p<0.01$. $^{***}p<0.001$.

ANCOVA analysis of covariance; *BID* twice daily; *BMI* body mass index; *GnRH* gonadotropin-releasing hormone; *NMPP* non-menstrual pelvic pain; *NSAID* non-steroidal anti-inflammatory drug; *QD* once daily.

Supplemental Table 5. Endometriosis Health Profile-30 at Month 3 – self-image

Variable	*N*	Least squares means (SE)	Difference of least squares means (SE)	95% CI	*p* value[b]	Treatment by subgroup *p* value[c]	Subgroup *p* value[c]
**Self-Image[a]**							
Age group (< 25 years, 25 to 35 years, > 35 years)						0.244	0.032*
Placebo	627	−10.22 (1.130)					
Elagolix 150 mg QD	417	−14.82 (1.364)	−4.59 (1.77)	(−8.07, −1.12)	0.010**		
Elagolix 200 mg BID	405	−20.37 (1.348)	−10.15 (1.76)	(−13.60, −6.70)	<0.001***		
BMI group (normal = < 25 kg/m^2, overweight = 25 to ≤ 29.9 kg/m^2, obese = ≥ 30 kg/m^2)						0.111	0.209
Placebo	625	−10.88 (0.933)					
Elagolix 150 mg QD	416	−16.36 (1.135)	−5.48 (1.47)	(−8.36, −2.59)	<0.001***		
Elagolix 200 mg BID	403	−21.32 (1.170)	−10.44 (1.50)	(−13.38, −7.51)	<0.001***		
Race group (White, Black, other)						0.783	0.294
Placebo	627	−11.65 (2.027)					
Elagolix 150 mg QD	417	−17.23 (2.607)	−5.58 (3.30)	(−12.05, 0.90)	0.091		
Elagolix 200 mg BID	405	−23.11 (2.734)	−11.46 (3.40)	(−18.13, −4.78)	<0.001***		
Ethnic group (Hispanic or Latino, other)						0.786	0.188
Placebo	627	−10.89 (1.297)					
Elagolix 150 mg QD	417	−15.31 (1.588)	−4.42 (2.05)	(−8.44, −0.39)	0.031*		
Elagolix 200 mg BID	405	−20.20 (1.582)	−9.31 (2.05)	(−13.32, −5.30)	<0.001***		
Baseline dysmenorrhea (< median = 2.17, ≥ median = 2.17)						0.144	<0.001***
Placebo	627	−11.16 (0.917)					
Elagolix 150 mg QD	417	−16.58 (1.124)	−5.42 (1.45)	(−8.26, −2.57)	<0.001***		
Elagolix 200 mg BID	405	−21.00 (1.142)	−9.84 (1.46)	(−12.71, −6.97)	<0.001***		
Baseline NMPP (< median = 1.54, ≥ median = 1.54)						0.473	<0.001***
Placebo	627	−11.15 (0.919)					
Elagolix 150 mg QD	417	−16.64 (1.127)	−5.49 (1.45)	(−8.35, −2.64)	<0.001***		
Elagolix 200 mg BID	405	−20.94 (1.147)	−9.80 (1.47)	(−12.68, −6.91)	<0.001***		
Baseline dyspareunia (< median = 1.40, ≥ median = 1.40)						0.611	0.040*
Placebo	513	−11.80 (1.021)					
Elagolix 150 mg QD	346	−16.82 (1.244)	−5.02 (1.61)	(−8.18, −1.87)	0.002**		

Elagolix 200 mg BID	326	−22.53 (1.281)	−10.74 (1.64)	(−13.95, −7.52)	<0.001***		
Time since endometriosis diagnosis (< 2 years, 2 to 5 years, ≥ 5 years)						0.604	0.002**
Placebo	627	−11.13 (0.921)					
Elagolix 150 mg QD	417	−16.98 (1.144)	−5.86 (1.47)	(−8.74, −2.97)	<0.001***		
Elagolix 200 mg BID	404	−21.09 (1.149)	−9.96 (1.47)	(−12.85, −7.07)	<0.001***		
Baseline analgesic use (none, opioid, NSAID, both)						0.088	0.183
Placebo	627	−10.17 (1.130)					
Elagolix 150 mg QD	417	−16.28 (1.247)	−6.11 (1.68)	(−9.41, −2.81)	<0.001***		
Elagolix 200 mg BID	405	−21.87 (1.412)	−11.70 (1.81)	(−15.24, −8.15)	<0.001***		
Previous GnRH therapy (yes, no)						0.604	0.978
Placebo	627	−10.77 (1.064)					
Elagolix 150 mg QD	417	−16.46 (1.291)	−5.69 (1.67)	(−8.97, −2.41)	<0.001***		
Elagolix 200 mg BID	405	−21.49 (1.290)	−10.72 (1.67)	(−14.00, −7.44)	<0.001***		
Entered washout (yes, no)						0.157	0.291
Placebo	627	−11.18 (1.042)					
Elagolix 150 mg QD	417	−15.05 (1.337)	−3.88 (1.70)	(−7.20, −0.55)	0.022*		
Elagolix 200 mg BID	405	−21.32 (1.324)	−10.15 (1.68)	(−13.45, −6.84)	<0.001***		
History of pregnancy at baseline (yes, no)						0.200	0.173
Placebo	627	−11.13 (0.931)					
Elagolix 150 mg QD	417	−16.57 (1.152)	−5.45 (1.48)	(−8.35, −2.54)	<0.001***		
Elagolix 200 mg BID	405	−20.75 (1.156)	−9.62 (1.48)	(−12.54, −6.71)	<0.001***		

[a]Each dimension for the core and modular questionnaire is calculated on a scale from 0 = best possible health status to 100 = worst possible health status.

[b]*p* value for test of difference between each elagolix dose group and placebo at each post-baseline time point is from an ANCOVA model with treatment as the main effect, baseline value, subgroup, and treatment × subgroup as covariates.

[c]*p* values are obtained from an ANCOVA model: change from baseline = treatment + baseline value + subgroup + treatment × subgroup.

**p*<0.05. ***p*<0.01. ****p*<0.001.

ANCOVA analysis of covariance; *BID* twice daily; *BMI* body mass index; *GnRH* gonadotropin-releasing hormone; *NMPP* non-menstrual pelvic pain; *NSAID* non-steroidal anti-inflammatory drug; *QD* once daily.

Supplemental Table 6. Endometriosis Health Profile-30 at month 3 – sexual intercourse

Variable	*N*	Least squares means (SE)	Difference of least squares means (SE)	95% CI	*p* value[b]	Treatment by subgroup *p* value[c]	Subgroup *p* value[c]
**Sexual Intercourse[a]**							
Age group (< 25 years, 25 to 35 years, > 35 years)						0.916	0.005**
Placebo	449	−13.43 (1.329)					
Elagolix 150 mg QD	306	−17.24 (1.585)	−3.80 (2.07)	(−7.86, 0.25)	0.066		
Elagolix 200 mg BID	279	−25.91 (1.610)	−12.47 (2.09)	(−16.57, −8.38)	<0.001***		
BMI group (normal = < 25 kg/m^2, overweight = 25 to ≤ 29.9 kg/m^2, obese = ≥ 30 kg/m^2)						0.359	0.427
Placebo	448	−13.48 (1.092)					
Elagolix 150 mg QD	305	−17.39 (1.328)	−3.91 (1.72)	(−7.28, −0.53)	0.023*		
Elagolix 200 mg BID	277	−26.29 (1.413)	−12.81 (1.79)	(−16.31, −9.30)	<0.001***		
Race group (White, Black, other)						0.005**	0.893
Placebo	449	−10.29 (2.811)					
Elagolix 150 mg QD	306	−16.23 (3.439)	−5.95 (4.44)	(−14.66, 2.77)	0.181		
Elagolix 200 mg BID	279	−32.03 (3.013)	−21.74 (4.12)	(−29.82, −13.66)	<0.001***		
Ethnic group (Hispanic or Latino, other)						0.242	0.909
Placebo	449	−13.31 (1.516)					
Elagolix 150 mg QD	306	−15.65 (1.849)	−2.34 (2.39)	(−7.03, 2.36)	0.329		
Elagolix 200 mg BID	279	−27.36 (1.880)	−14.04 (2.42)	(−18.78, −9.30)	<0.001***		
Baseline dysmenorrhea (< median = 2.17, ≥ median = 2.17)						0.388	0.089
Placebo	449	−13.54 (1.080)					
Elagolix 150 mg QD	306	−17.34 (1.310)	−3.80 (1.70)	(−7.14, −0.47)	0.025*		
Elagolix 200 mg BID	279	−25.81 (1.370)	−12.27 (1.74)	(−15.69, −8.85)	<0.001***		
Baseline NMPP (< median = 1.54, ≥ median = 1.54)						0.111	0.160
Placebo	449	−13.54 (1.079)					
Elagolix 150 mg QD	306	−17.42 (1.310)	−3.88 (1.70)	(−7.21, −0.55)	0.023*		
Elagolix 200 mg BID	279	−25.77 (1.374)	−12.23 (1.75)	(−15.66, −8.80)	<0.001***		
Baseline dyspareunia (< median = 1.40, ≥ median = 1.40)						0.649	0.007**
Placebo	437	−13.65 (1.092)					
Elagolix 150 mg QD	296	−17.45 (1.327)	−3.81 (1.72)	(−7.18, −0.44)	0.027*		

Elagolix 200 mg BID	271	−26.08 (1.387)	−12.43 (1.76)	(−15.90, −8.97)	<0.001***		
Time since endometriosis diagnosis (< 2 years, 2 to < 5 years, ≥ 5 years)						0.213	0.668
Placebo	449	−13.61 (1.081)					
Elagolix 150 mg QD	306	−17.62 (1.337)	−4.02 (1.72)	(−7.39, −0.64)	0.020*		
Elagolix 200 mg BID	279	−25.83 (1.373)	−12.22 (1.75)	(−15.65, −8.79)	<0.001***		
Baseline analgesic use (none, opioids, NSAID, both)						0.016*	0.180
Placebo	449	−13.35 (1.305)					
Elagolix 150 mg QD	306	−16.66 (1.428)	−3.31 (1.93)	(−7.10, 0.49)	0.088		
Elagolix 200 mg BID	279	−28.00 (1.861)	−14.64 (2.27)	(−19.11, −10.18)	<0.001***		
Previous GnRH therapy (yes, no)						0.074	0.232
Placebo	449	−13.02 (1.236)					
Elagolix 150 mg QD	306	−15.52 (1.498)	−2.50 (1.94)	(−6.31, 1.31)	0.198		
Elagolix 200 mg BID	279	−26.64 (1.602)	−13.63 (2.02)	(−17.59, −9.66)	<0.001***		
Entered washout (yes, no)						0.436	0.677
Placebo	449	−13.13 (1.235)					
Elagolix 150 mg QD	306	−17.30 (1.559)	−4.17 (1.99)	(−8.07, −0.27)	0.036*		
Elagolix 200 mg BID	279	−26.81 (1.631)	−13.68 (2.05)	(−17.70, −9.67)	<0.001***		
History of pregnancy at baseline (yes, no)						0.010**	0.006**
Placebo	449	−13.63 (1.119)					
Elagolix 150 mg QD	306	−16.86 (1.346)	−3.22 (1.75)	(−6.66, 0.21)	0.066		
Elagolix 200 mg BID	279	−24.59 (1.407)	−10.95 (1.80)	(−14.48, −7.43)	<0.001***		

[a]Each dimension for the core and modular questionnaire is calculated on a scale from 0 = best possible health status to 100 = worst possible health status.
[b]*p* value for test of difference between each elagolix dose group and placebo at each post-baseline time point is from an ANCOVA model with treatment as the main effect, baseline value, subgroup, and treatment × subgroup as covariates.
[c]*p* values are obtained from an ANCOVA model: change from baseline = treatment + baseline value + subgroup + treatment × subgroup.
$^{*}p<0.05$. $^{**}p<0.01$. $^{***}p<0.001$.
ANCOVA analysis of covariance; *BID* twice daily; *BMI* body mass index; *GnRH* gonadotropin-releasing hormone; *NMPP* non-menstrual pelvic pain; *NSAID* non-steroidal anti-inflammatory drug; *QD* once daily.

Supplemental Table 7. Adverse events from integrated Elaris EM-I and Elaris EM-II by selected subgroup[a]

	Any AE						Any serious AE			Any severe AE			Any AE leading to discontinuation		
**Variable**	**Placebo**		**150 mg ELA QD**		**200 mg ELA BID**		**Placebo**	**150 mg ELA QD**	**200 mg ELA BID**	**Placebo**	**150 mg ELA QD**	**200 mg ELA BID**	**Placebo**	**150 mg ELA QD**	**200 mg ELA BID**
	N		***N***		***N***										
Age (years)															
< 25	95	67 (70.5)	58	42 (72.4)	66	57 (86.4)	5 (5.3)	2 (3.4)	1 (1.5)	8 (8.4)	4 (6.9)	12 (18.2)	3 (3.2)	2 (3.4)	7 (10.6)
25−29	164	125 (76.2)	104	88 (84.6)	106	85 (80.2)	6 (3.7)	3 (2.9)	4 (3.8)	23 (14.0)	13 (12.5)	15 (14.2)	16 (9.8)	4 (3.8)	8 (7.5)
30−34	212	147 (69.3)	153	120 (78.4)	140	117 (83.6)	5 (2.4)	5 (3.3)	4 (2.9)	29 (13.7)	19 (12.4)	16 (11.4)	9 (4.2)	14 (9.2)	12 (8.6)
35−39	143	112 (78.3)	91	74 (81.3)	86	73 (84.9)	3 (2.1)	1 (1.1)	2 (2.3)	16 (11.2)	8 (8.8)	10 (11.6)	4 (2.8)	4 (4.4)	10 (11.6)
≥ 40	120	86 (71.7)	69	56 (81.2)	79	67 (84.8)	5 (4.2)	3 (4.3)	1 (1.3)	12 (10.0)	5 (7.2)	11 (13.9)	12 (10.0)	2 (2.9)	9 (11.4)
Time Since Diagnosis (months)															
< 48	449	320 (71.3)	307	240 (78.2)	285	234 (82.1)	17 (3.8)	5 (1.6)	6 (2.1)	52 (11.6)	32 (10.4)	39 (13.7)	27 (6.0)	15 (4.9)	31 (10.9)
≥ 48	285	217 (76.1)	168	140 (83.3)	191	164 (85.9)	7 (2.5)	9 (5.4)	6 (3.1)	36 (12.6)	17 (10.1)	24 (12.6)	17 (6.0)	11 (6.5)	15 (7.9)
Geographic Region															
US	548	397 (72.4)	355	285 (80.3)	358	293 (81.8)	16 (2.9)	7 (2.0)	9 (2.5)	70 (12.8)	36 (10.1)	50 (14.0)	33 (6.0)	22 (6.2)	33 (9.2)
Non-US	186	140 (75.3)	120	95 (79.2)	119	106 (89.1)	8 (4.3)	7 (5.8)	3 (2.5)	18 (9.7)	13 (10.8)	14 (11.8)	11 (5.9)	4 (3.3)	13 (10.9)
History of Depression															
Yes	133	17 (12.8)	88	13 (14.8)	103	20 (19.4)	0 (0)	0 (0)	0 (0)	1 (0.8)	1 (1.1)	2 (1.9)	1 (0.8)	1 (1.1)	3 (2.9)
No	601	28 (4.7)	387	31 (8.0)	374	39 (10.4)	0 (0)	1 (0.3)	0 (0)	1 (0.2)	1 (0.3)	4 (1.1)	4 (0.7)	3 (0.8)	7 (1.9)

[a]Adverse events were coded using the Medical Dictionary for Regulatory Activities, versions 18.0 (Elaris EM-I) and 19.0 (Elaris EM-II). The severity of each AE was rated by the investigator as mild, moderate, or severe. Serious AEs were defined as life-threatening, requiring hospitalization or medical or surgical intervention to prevent a serious outcome, or resulting in persistent disability or death. One death occurred in the subgroup—no history of depression, US based, ≥ 48 months since diagnosis, ≥ 40 years old taking elagolix 150 mg once daily. *AE* adverse event; *BID* twice daily; *ELA* elagolix; *QD* once daily.

Supplemental Figure Legends

Supplemental Fig. 1 Co-primary endpoints at month 3 for integrated Elaris EM-I and II by baseline demographic subgroups. (A) Dysmenorrhea responders. (B) Non-menstrual pelvic pain responders. *$p<0.05$, **$p<0.01$, and ***$p<0.001$ indicate comparison vs. placebo based on a logistic regression model. *BID* twice daily; *BMI* body mass index; *QD* once daily.

Supplemental Fig. 2 Co-primary endpoints at month 3 for integrated Elaris EM-I and Elaris EM-II by disease severity subgroups. (A) Dysmenorrhea responders. (B) Non-menstrual pelvic pain (NMPP) responders. *$p<0.05$, **$p<0.01$, and ***$p<0.001$ indicate comparison vs. placebo based on a logistic regression model. *BID* twice daily; *GnRH* gonadotropin-releasing hormone; *NSAID* non-steroidal anti-inflammatory drug; *QD* once daily.

Supplemental Fig. 3 Patient Global Impression of Change (PGIC) at month 3 for integrated Elaris EM-I and Elaris EM-II by baseline demographic subgroups. *$p<0.05$. **$p<0.01$. ***$p<0.001$. *BID* twice daily; *BMI* body mass index; *QD* once daily.

Supplemental Fig. 4 Patient Global Impression of Change (PGIC) at month 3 for integrated Elaris EM-I and Elaris EM-II by disease severity subgroups. *$p<0.05$. **$p<0.01$. ***$p<0.001$. *BID* twice daily; *GnRH* gonadotropin-releasing hormone; *NMPP* non-menstrual pelvic pain; *QD* once daily.

SUPPLEMENTAL FIGURES

Supplemental Figure 1A

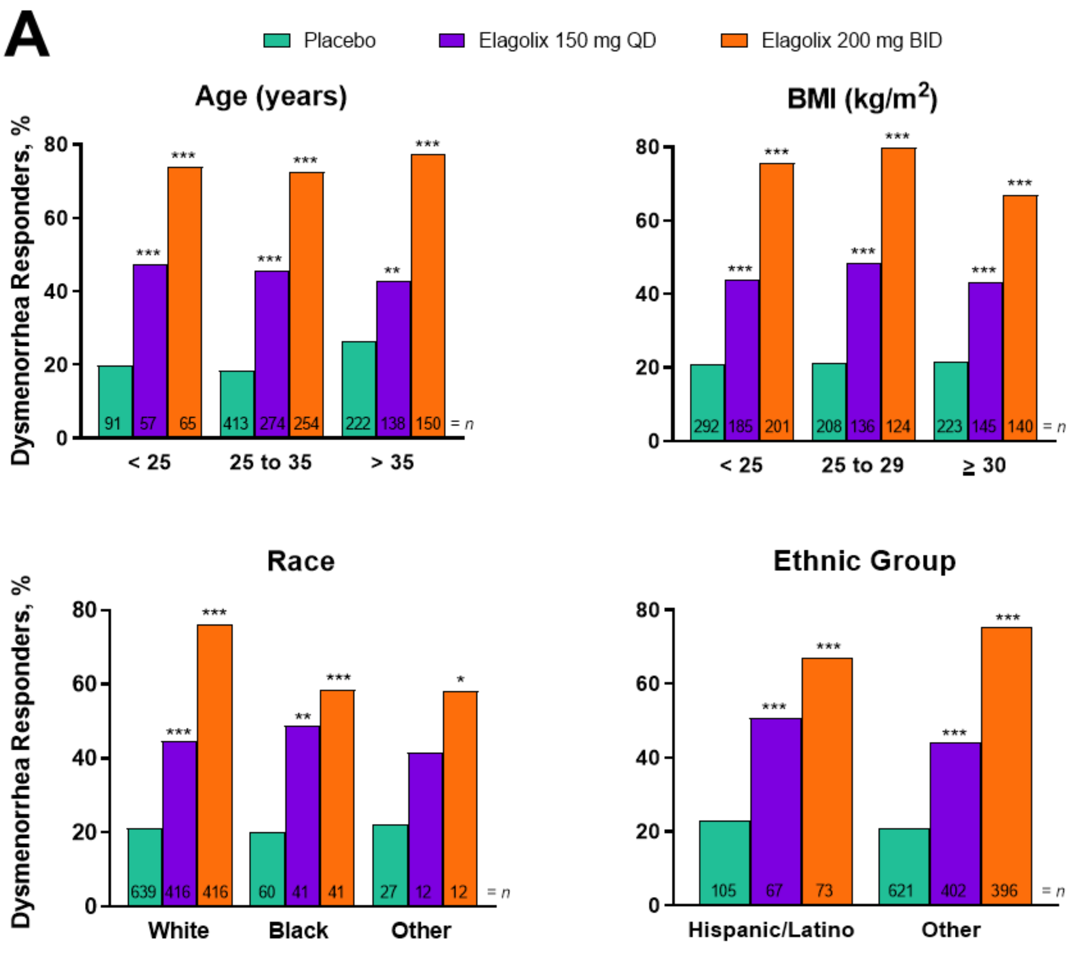

Supplemental Figure 1B

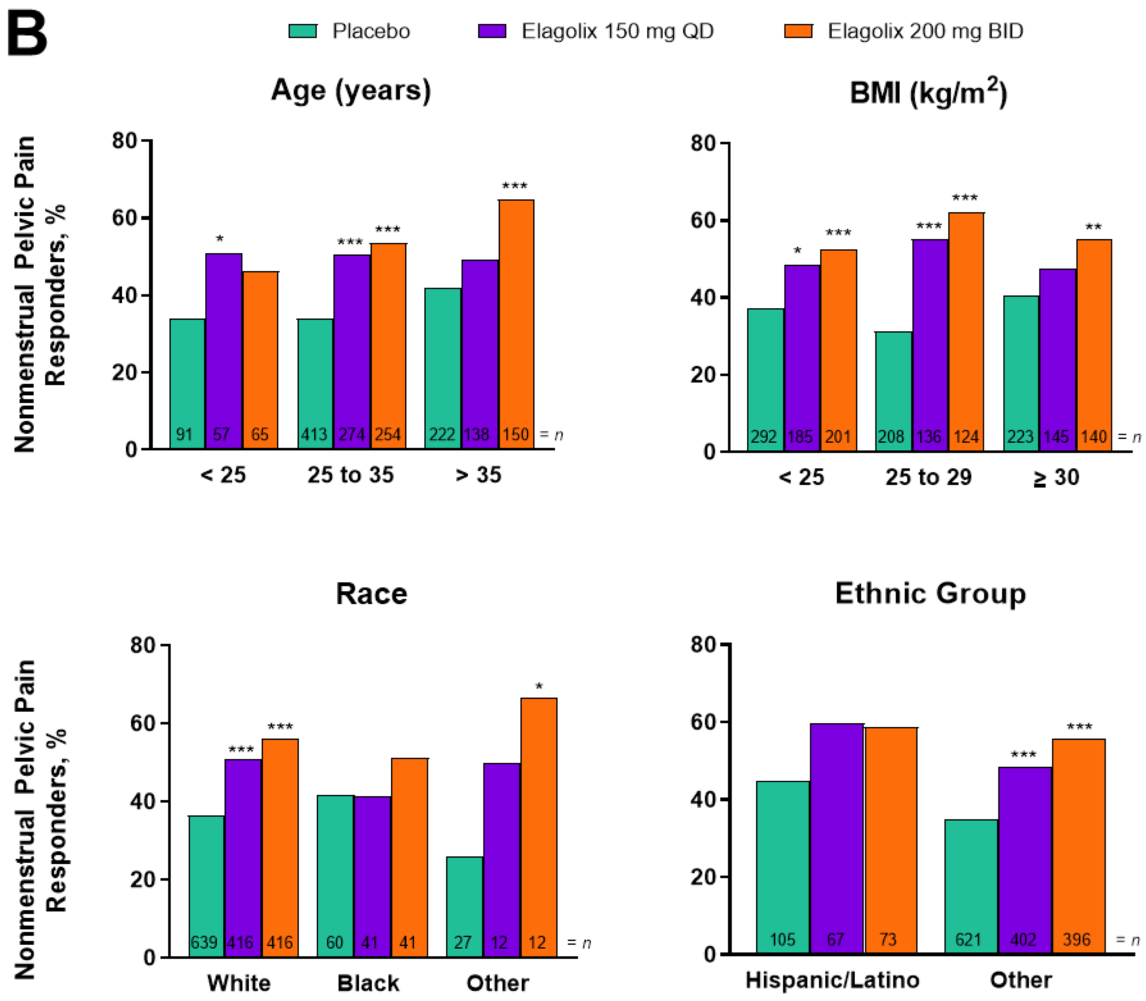

Supplemental Figure 2A

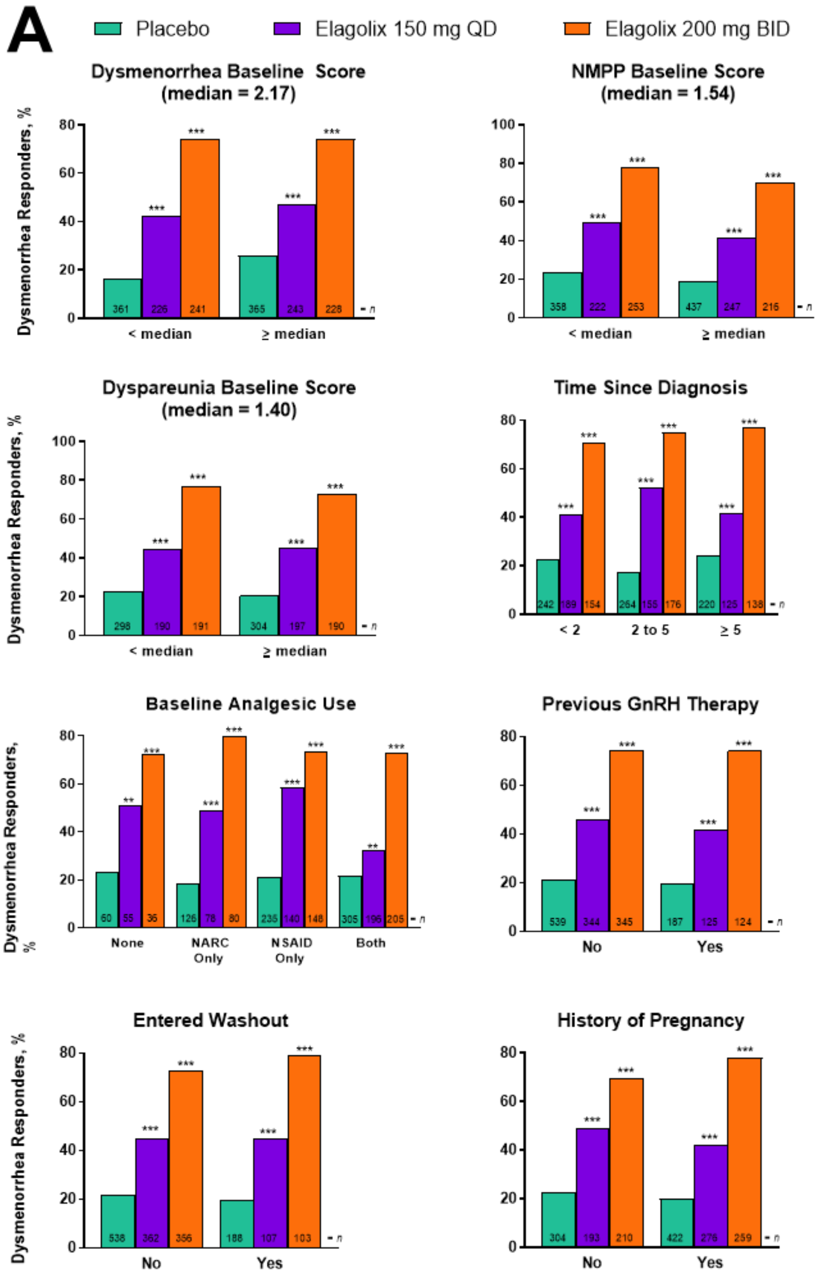

Supplemental Figure 2B

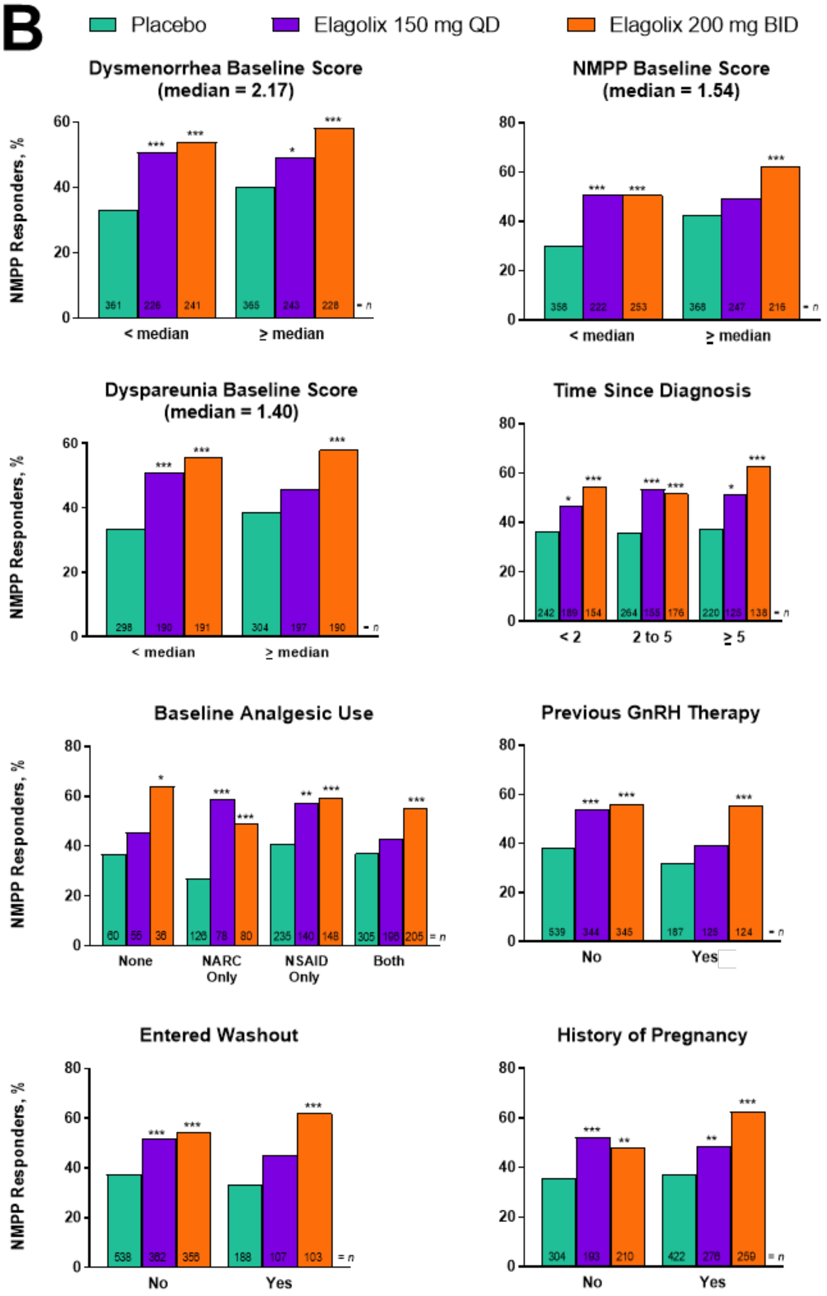

Supplemental Figure 3

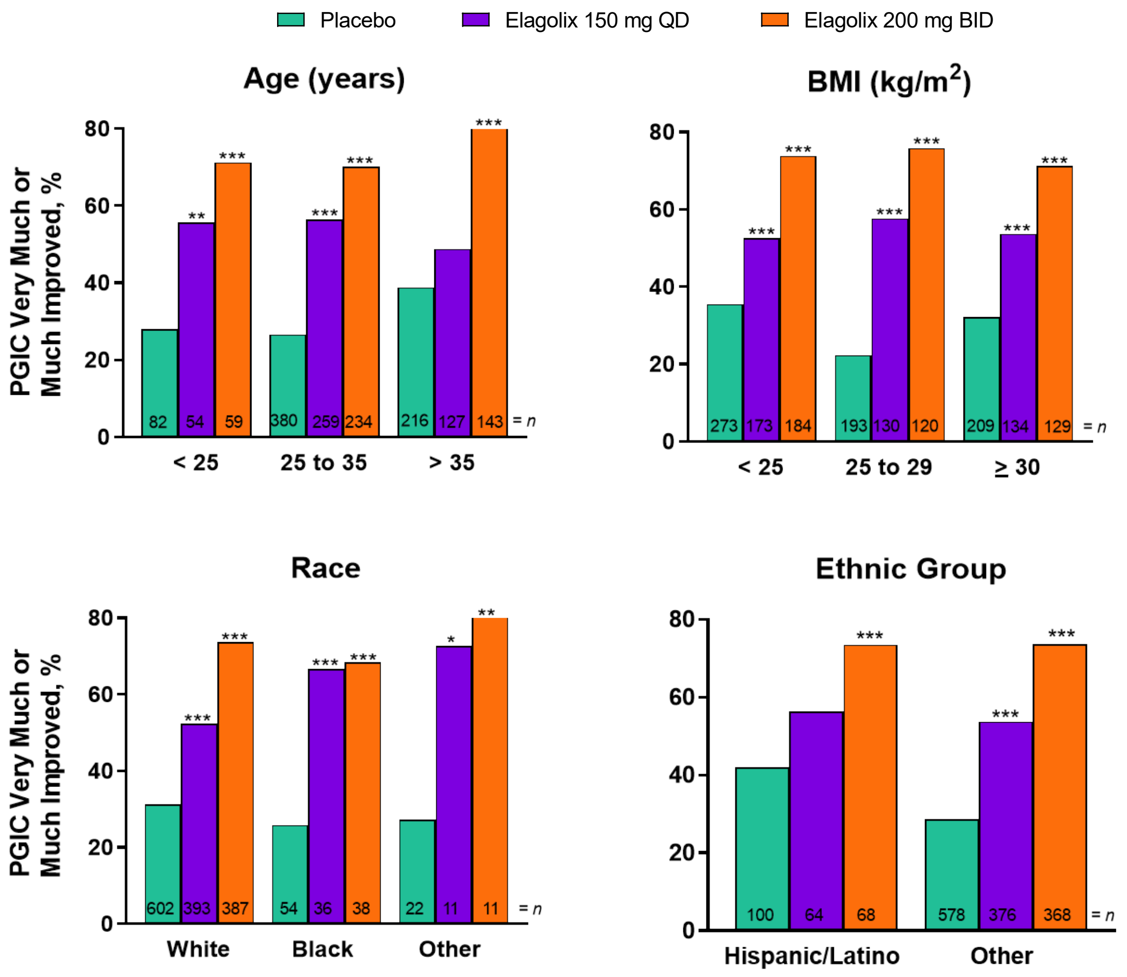

Supplemental Figure 4

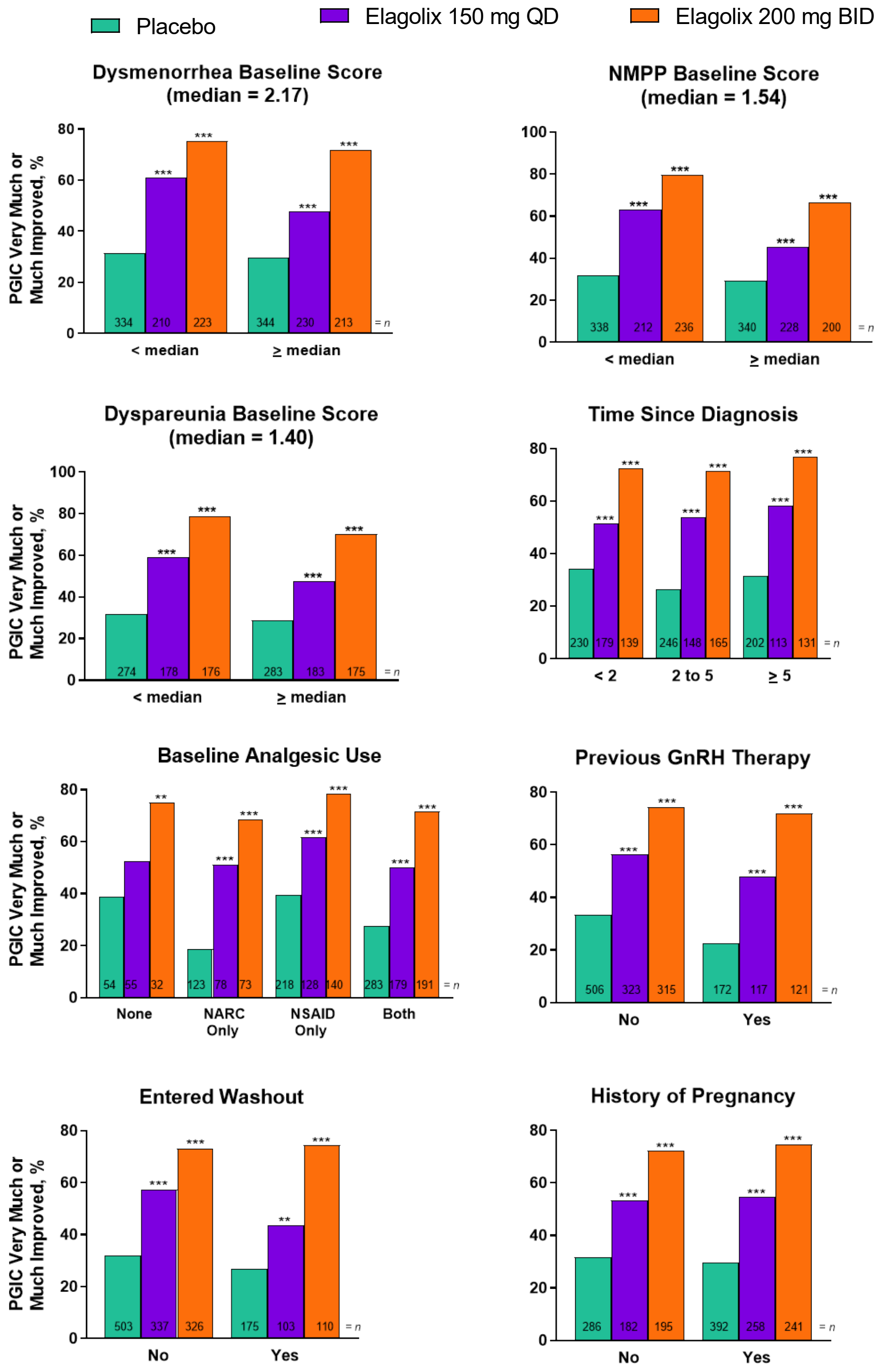

Supplement: Supplementary file 1 — Additional file 1: Supplemental Table 1. Endometriosis Health Profile-30 at month 3 – pain; Supplemental Table 2. Endometriosis Health Profile-30 at month 3 – control and powerlessness; Supplemental Table 3. Endometriosis Health Profile-30 at month 3 – emotional well-being; Supplemental Table 4. Endometriosis Health Profile-30 at month 3 – social support; Supplemental Table 5. Endometriosis Health Profile-30 at Month 3 – self-image; Supplemental Table 6. Endometriosis Health Profile-30 at month 3 – sexual intercourse; Supplemental Table 7. Adverse events from integrated Elaris EM-I and Elaris EM-II by selected subgroup; Supplemental Figure 1. Co-primary endpoints at month 3 for integrated Elaris EM-I and II by baseline demographic subgroups; Supplemental Figure 2. Co-primary endpoints at month 3 for integrated Elaris EM-I and Elaris EM-II by disease severity subgroups; Supplemental Figure 3. Patient Global Impression of Change (PGIC) at month 3 for integrated Elaris EM-I and Elaris EM-II by baseline demographic subgroups; Supplemental Figure 4. Patient Global Impression of Change (PGIC) at month 3 for integrated Elaris EM-I and Elaris EM-II by disease severity subgroups. [file 12905_2021_1385_MOESM1_ESM.pdf]
